# Remodulation of bacterial transcriptome after acquisition of foreign DNA: the case of *irp*-HPI high-pathogenicity island in *Vibrio anguillarum*

**DOI:** 10.1128/msphere.00596-23

**Published:** 2023-12-11

**Authors:** Marta A. Lages, Ana do Vale, Manuel L. Lemos, Miguel Balado

**Affiliations:** 1 Department of Microbiology and Parasitology, Institute of Aquaculture, University of Santiago de Compostela, Santiago de Compostela, Spain; 2 Fish Immunology and Vaccinology Group, i3S-Instituto de Investigação e Inovação em Saúde, Universidade do Porto, Porto, Portugal; University of Kentucky College of Medicine, Lexington, Kentucky, USA

**Keywords:** *Vibrio anguillarum*, virulence factors, transcriptome analysis, genomic island, horizontal gene transfer

## Abstract

The high-pathogenicity island *irp*-HPI is widespread in *Vibrionaceae* and encodes the siderophore piscibactin, as well as the regulator PbtA that is essential for its expression. In this work, we aim to study whether PbtA directly interacts with *irp*-HPI promoters. Furthermore, we hypothesize that PbtA, and thereby the acquisition of *irp*-HPI island, may also influence the expression of other genes elsewhere in the bacterial genome. To address this question, an RNAseq analysis was conducted to identify differentially expressed genes after *pbtA* deletion in *Vibrio anguillarum* RV22 genetic background. The results showed that PbtA not only modulates the *irp*-HPI genes but also modulates the expression of a plethora of *V. anguillarum* core genome genes, inducing nitrate, arginine, and sulfate metabolism, T6SS1, and quorum sensing, while repressing lipopolysaccharide (LPS) production, MARTX toxin, and major porins such as OmpV and ChiP. The direct binding of the C-terminal domain of PbtA to piscibactin promoters (P*frpA* and P*frpC*), quorum sensing (*vanT*), LPS transporter *wza,* and T6SS structure- and effector-encoding genes was demonstrated by electrophoretic mobility shift assay (EMSA). The results provide valuable insights into the regulatory mechanisms underlying the expression of *irp*-HPI island and its impact on Vibrios transcriptome, with implications in pathogenesis.

**IMPORTANCE:**

Horizontal gene transfer enables bacteria to acquire traits, such as virulence factors, thereby increasing the risk of the emergence of new pathogens. *irp*-HPI genomic island has a broad dissemination in *Vibrionaceae* and is present in numerous potentially pathogenic marine bacteria, some of which can infect humans. Previous works showed that certain *V. anguillarum* strains exhibit an expanded host range plasticity and heightened virulence, a phenomenon linked to the acquisition of the *irp*-HPI genomic island. The present work shows that this adaptive capability is likely achieved through comprehensive changes in the transcriptome of the bacteria and that these changes are mediated by the master regulator PbtA encoded within the *irp*-HPI element. Our results shed light on the broad implications of horizontal gene transfer in bacterial evolution, showing that the acquired DNA can directly mediate changes in the expression of the core genome, with profounds implications in pathogenesis.

## INTRODUCTION

Horizontal gene transfer (HGT) enables bacteria to acquire beneficial traits such as niche colonization and symbiosis capacities, antimicrobial resistance, or virulence factors (1–3). The introgression of concrete transferable DNA elements into bacterial populations, such as the “Cholera Toxin Phage” (CTXφ) in *Vibrio cholerae* or pGV1512 plasmid in *Vibrio crassostreae*, is directly associated with the emergence of novel pathogens (4, 5). However, the newly acquired genes can impose an excessive fitness cost on recipient bacteria caused by, e.g., their uncontrolled expression (6). To counterbalance these effects, bacteria have evolved mechanisms, known as xenogeneic silencers, to repress the expression of horizontally acquired DNA (7). Interestingly, DNA acquired through horizontal gene transfer usually encodes factors that ensure its own expression *ad hoc* (8). Thus, the acquired DNA must be integrated into the recipient genome regulatory network.

The *Vibrio* genus is characterized by a large genotypic diversity, which is partially attributed to HGT (9). This encompasses a wide range of niche specialization from free-living bacteria to those associated with other organisms in a mutualistic, commensal, or pathogenic relationship (9). *Vibrio anguillarum* is able to cause hemorrhagic septicemia (vibriosis) in warm- and cold-water fish species, leading to high mortalities and economic losses in aquaculture worldwide (10, 11). Several virulence-related factors have been identified in this species, including lipopolysaccharide (LPS) (the most virulent strains belong to serotypes O1, O2, and, to a lesser extent, O3), motility and chemotaxis, quorum sensing, extracellular products with hemolytic and proteolytic activities, and up to three siderophores (11–13). Notably, certain *V. anguillarum* strains exhibit high virulence in a broad host range, causing great mortality rates in warm- and cold-water-adapted fish, even at temperatures as low as 7°C, which is well below the bacterial optimal growth conditions (14, 15). This expanded virulence phenotype has been associated with the acquisition of the high-pathogenicity island *irp*-HPI through HGT (16, 17).

**FIG 1:**
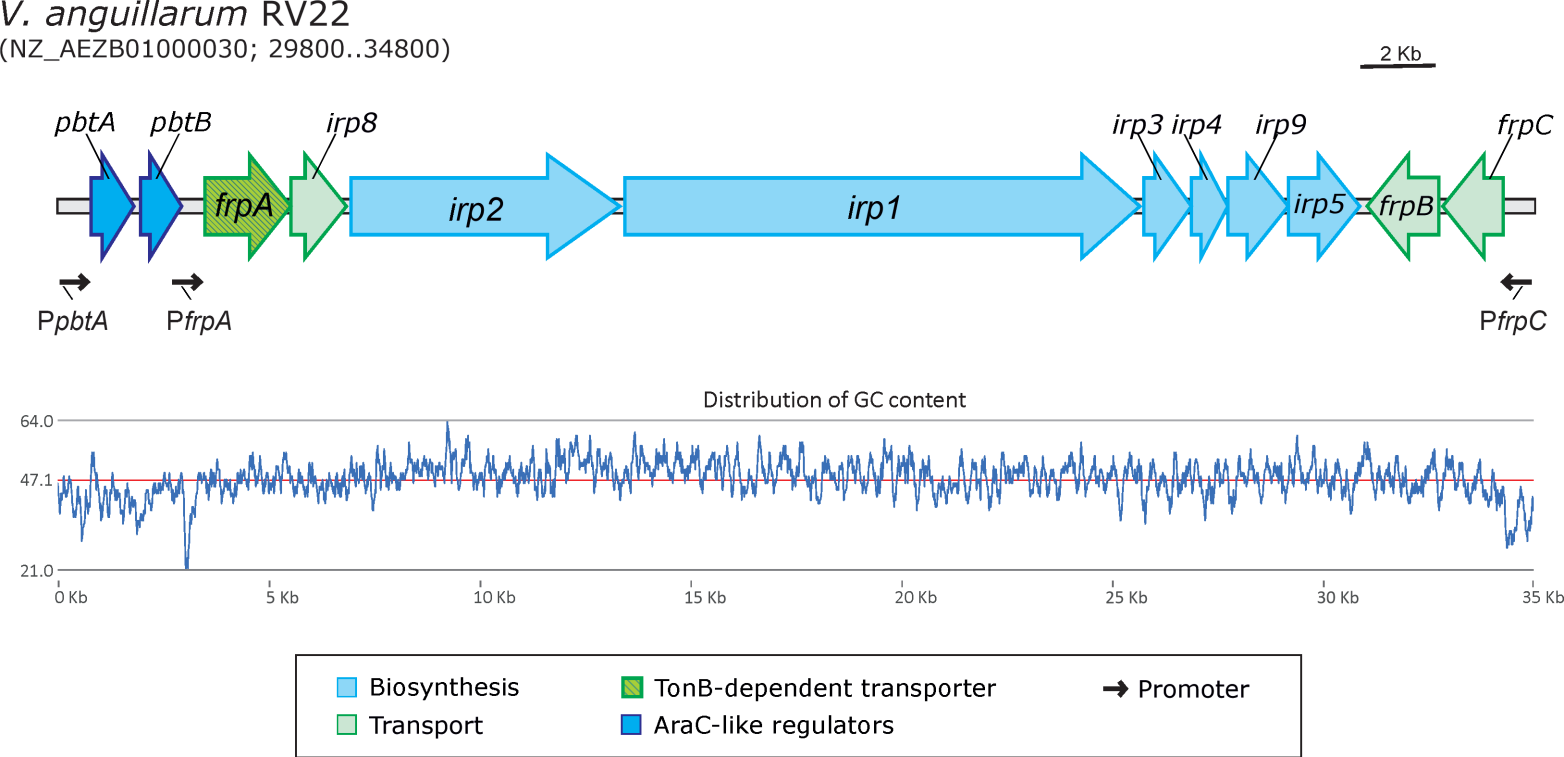
Genetic map and distribution of GC content of *irp*-HPI genomic island encoding piscibactin system in *Vibrio anguillarum*.

The *irp*-HPI genomic island harbors the *irp* genes, which are responsible for the synthesis, transport, and utilization of the siderophore piscibactin (18, 19) (Fig. 1). Piscibactin production plays a key role in virulence, not only in *V. anguillarum* (16) but also in other fish or mollusc pathogens such as *Photobacterium damselae* subsp. *piscicida* (20), *V. ordalii* (21), and *V. neptunius* (22). *irp*-HPI has a broad dissemination in *Vibrionaceae* and is present in numerous potentially pathogenic marine bacteria, some of which can infect humans (22–24). The piscibactin system has a dual requirement for iron starvation and low temperature to be expressed and is one of the most induced virulence factors in *V. anguillarum* when growth temperature drops below 20°C (16). Our recent work showed that the expression of piscibactin is controlled via a regulatory cascade involving the global regulators H-NS and ToxR-S, though none of them is the main actor (24). The *irp*-HPI encodes an AraC-like transcriptional regulator named PbtA (Fig. 1), whose inactivation disables the expression of piscibactin biosynthesis and transport genes (24, 25). PbtA expression is greatly induced with temperature decrease, making it the main activator of the piscibactin siderophore system expression under low temperature. Interestingly, PbtA deletion results in a dramatic decrease in the degree of virulence, a decrease higher than that found after the inactivation of the piscibactin system alone (24).

In this study, we aimed to investigate whether the transcriptional regulator PbtA, and thereby the acquisition of the *irp*-HPI island, may influence the expression of core genes in *V. anguillarum*. To this purpose, an RNAseq analysis was performed to identify differentially expressed genes (DEGs) after the inactivation of *pbtA* in *V. anguillarum* RV22, a highly pathogenic strain that harbors the *irp*-HPI island (26)(16). Moreover, PbtA C- and N-terminal domains were purified, and their binding ability to the *irp*-HPI promoters and to promoters of some DEGs was tested by gel mobility shift analysis. Thus, *V. anguillarum* was used as a model system to elucidate the interaction of the *irp*-HPI island with the genome of the recipient bacteria. Our results provide valuable insights into the regulatory mechanisms underlying the expression of the horizontally acquired *irp*-HPI island and its impact on *Vibrio* transcriptome.

## EXPERIMENTAL PROCEDURES

### PbtA *in silico* analysis, recombinant protein expression, and purification

UniProt (27) and BLAST (28) were used to perform the functional and homology analysis of *V. anguillarum* PbtA (UniProtKB accession: A0A289GIJ0). PbtA and its N- or C-terminal domains (PbtA^N^ and PbtA^C^, respectively) were cloned into the expression vector pET20b(+). To obtain the PbtA^N^ fusion protein, the region encoding amino acids M1-E211 was PCR amplified, whereas to create the PbtA^C^ fusion protein, the region encoding amino acids S213-P326 was PCR amplified and cloned into the *NdeI* and *XhoI* restriction sites of the pET20b(+) vector in frame with a C-terminal (PbtA and PbtA^N^) or N-terminal His-tag (PbtA^C^). The oligonucleotides used are listed in Table S1.

The *E. coli* expression strains (Table S2) and the induction temperature were selected based on expression tests performed to determine the optimal conditions to achieve the maximum protein yield. Therefore, PbtA and PbtA^N^ were overexpressed in *E. coli* BL21 at 17°C and 37°C, respectively, whereas PbtA^C^ was expressed in *E. coli* BL21 pLysS at 37°C. The correspondent *E. coli* cells carrying the expression vectors were grown aerobically in LB medium at 17°C or 37°C until an OD_600_ of 0.5 was reached, and protein expression was induced by adding 0.5 mM of IPTG and carried out overnight. Induced cells were collected by centrifugation at 4,000 × *g* for 30 min at 4°C, the supernatant was discarded, and the pellet was resuspended in 40 mL of resuspension buffer (50 mM Tris-HCl pH 8.0, 500 mM NaCl). The cells were lysed by sonication, and the lysate was centrifuged at 35,000 × *g* for 30 min at 4°C. The resultant supernatant was loaded onto a C-50 column packed with high-density nickel resin (ABT), and the fusion proteins were step-eluted with 50 mM Tris-HCl, pH 8.0, and 500 mM NaCl containing increasing concentrations of imidazole. The elution(s) containing the desired protein was concentrated at 4°C using Vivaspin centrifugal concentrators following the manufacturer’s instructions and dialyzed (Spectra/Por dialysis membrane, Spectrum Labs) against 1 L of 50 mM Tris-HCl, pH 8.0, and 500 mM NaCl overnight at 4°C. The purified proteins were analyzed by SDS-PAGE, and the concentration was determined using a NanoDrop ND-1000 Spectrophotometer, taking into account the extinction coefficient (at 280 nm), and the molecular weight was calculated using ProtParam (29). The proteins were stored at −80°C until further use.

### Electrophoretic mobility shift assay (EMSA)

The DNA regions to be tested were PCR amplified and end-labeled using the Biotin 3′ End-Labeling Kit (Thermo Scientific) following the manufacturer’s instructions. EMSAs were performed using the LightShift Chemiluminescent EMSA Kit (Thermo Scientific) following supplier’s recommendations. Briefly, 0.2 pmol, 0.5 pmol, or 1 pmol of PbtA^N^ or PbtA^C^ were mixed with 25 fmol of biotin-labeled DNA in a binding buffer containing 1 mM Tris, pH 7.5, 155 mM KCl, 0.1 mM DTT, 5% glycerol, 5 mM MgCl_2_, 1 mM EDTA, and 0.3 mg/mL BSA. To ensure the specificity of the interaction, a 100-fold molar excess of specific unlabeled DNA was added as a control. Binding reactions were incubated for 30 min at 20°C, loaded on a 5% polyacrylamide gel (acrylamide:bis-acrylamide, 29:1) in 0.5 × Tris Borate EDTA buffer, electrophoresed at 100 V for 1 h at 4°C and then transferred to a positively charged nylon membrane. The transferred DNA was crosslinked to the membrane using a UV-light Linus MiniCross equipment. Biotin-labeled DNA was detected by chemiluminescence using a Fujifilm LAS-3000. The intensity of the bands was quantified using Multi-Gauge v 3.0 software (Fujifilm), and relative quantification of the band was calculated as the ratio to labeled probe controls. At least four relative quantifications were performed per EMSA band. Statistical significance was determined by Student’s *t* test with a threshold *P* value < 0.05.

### Construction of *pbtA* and *pbtA^N^* defective mutants and complementation

In-frame deletions of *pbtA* and *pbtA^N^* were constructed by allelic exchange in *V. anguillarum* RV22 wild-type strain background, respectively. The flanking sequences of the gene region encoding the N-terminal domain of PbtA (PbtA^N^) were PCR amplified and cloned into the low-copy number vector pWKS30 (30). The plasmid was digested with NotI and ApaI, and the allele was cloned into the suicide vector pNidKan (31), resulting in the creation of the plasmid named pML1287. Subsequently, the previously constructed plasmid pML118, used for generating the Δ*pbtA* defective mutant (Table S2), and pML1287 were conjugated with the correspondent *V. anguillarum* strain, and transconjugants selection was based on ampicillin and kanamycin resistance. After a second event of recombination, the mutant strain was selected based on sucrose (15%) resistance and subsequent growth on ampicillin and kanamycin plates. PCR was used to confirm the allelic exchange event. Mutant complementation was achieved by cloning the wild-type *pbtA* gene into the vector pSEVA651 (32) in *E. coli* S17-1 λpir. The plasmid was then mobilized to the mutant strain by conjugation.

### Transcriptional fusions and β-galactosidase assays

The previously constructed plasmids pMB276, pMB277, and pML212 (16, 25) carrying the *lacZ* fusions P*frpA::lacZ*, P*pbtA::lacZ,* and P*frpC::lacZ* (Fig. 1) into plasmid pPHRP309 (33) were mobilized from *E. coli* S17-1 λpir (34) to *V. anguillarum* RV22 and the derivative mutants Δ*pbtA* and Δ*pbtA^N^* by conjugation. The resultant constructions were confirmed by PCR amplification of the promoter regions. For the evaluation of the transcriptional activity, the *V. anguillarum* strains carrying the *lacZ* fusions were grown under weak iron-restrictive conditions in CM9 minimal medium supplemented with 25 µM 2,2′-dipyridyl. The bacterial cultures were grown aerobically at 15°C until they reached an OD600 = 0.3. β-galactosidase activities were measured following the method of Miller (35). The results shown are the mean of three independent experiments. Statistical significance was determined by Student’s *t* test with a threshold *P* value < 0.05.

### Growth conditions and total RNA extraction

*V. anguillarum* RV22 wild-type and RV22 Δ*pbtA* mutant strains were grown at 15°C in CM9 minimal medium under iron-restricted conditions achieved by adding 50 µM of 2,2′-μM dipyridyl. Bacterial cultures were grown until mid-exponential phase (OD_600_≈0.8) and harvested by centrifugation at 10,000 × *g* for 10 min. Total RNA from three independent cultures was isolated using TRIzol Reagent (Invitrogen) following the manufacturer’s instructions. RNA integrity was visualized in a 1% agarose gel, and the concentration was determined using Qubit TM RNA BR Assay Kit (Invitrogen). RNA was stored at −80°C until further use. The RNA integrity number (RIN) of the samples, measured using an Agilent 2100 Bioanalyzer, was higher than 8.

### cDNA library construction and sequencing

Triplicate biological samples were used for RNA extraction to create independent cDNA libraries for whole-transcriptome sequencing. Before library construction, residual DNA was eliminated and bacterial rRNA depletion was performed. Each biological replicate was represented by a separate library, consisting of approximately 20 million 2 × 150 bp reads. The sequencing was conducted on an Illumina MiSeq sequencing machine using the NextSeq High Output 1 × 150 pb kit. The construction of cDNA libraries and sequencing services were provided by the FISABIO Sequencing and Bioinformatics Service (Valencia, Spain, http://fisabio.san.gva.es/secuenciacion-masiva-y-bioinformatica). RNAseq reads per replicates were deposited at SRA database (Sequence Read Archive) under BioProject id. PRJNA991769 (Table S3).

### Bioinformatic analysis and gene expression quantification

The Tuxedo suite, a collection of open-source software programs (36), was utilized for RNAseq data analysis and validation. Briefly, Tophat was used to align the reads to *V. anguillarum* RV22 genome (GenBank acc. No. GCA_000257185.1). Reads mapped statistics are shown in Table S3. The mapped reads were then assembled into potential transcripts, and a final transcriptome assembly was generated using Cufflinks. Cuffdiff was used for the differential expression analysis of genes across wild-type and *pbtA* mutant strains. The output files from Cuffdiff were processed using the R package CummeRbund, generating volcano plot and figures. Functional classification of differentially expressed genes (DEGs) was performed using the Kyoto Encyclopedia of Genes and Genomes (KEGG) database.

### Reverse Transcription-Quantitative PCR (RT-qPCR) validation

The RNA concentration of each sample was adjusted to 10 µg/µL, and 4 µg was subjected to DNaseI treatment to ensure the total removal of DNA. The resultant RNA was used for the quantitative analysis using One-step NZY RT-qPCR Green kit (NZYTech). Primers used to detect the expression of 16S ribosomal RNA (reference gene), *frpA*, *wza*, *hcp1*, and *vanT* are listed in Table S1. Three independent RT-qPCR reactions were performed using a CFX96 Real-Time PCR Detection System (Bio-Rad) following the cycling setup with an initial reverse transcription of 50°C for 20 min, polymerase activation at 95°C for 10 min, and finally, 40 cycles of denaturation at 95°C for 15 s and annealing/extension at 60°C for 30 s. To confirm the amplification of only one product, a melting curve was performed from 65°C to 95°C with consecutive increments of 0.5°C each 5 s. The 2^(-ΔΔct)^ method (37) was used to calculate the relative fold gene expression normalized with rRNA 16S.

### Biofilm formation

The crystal violet staining assay was used for biofilm quantification. *V. anguillarum* RV22, Δ*pbtA* mutant, and the correspondent complemented strain were grown overnight aerobically at 25°C. The cultures were adjusted to an OD_600_ of 0.5, and a final dilution of 1:20 was inoculated in CM9 minimal medium supplemented with 50 µM of 2,2′-dipyridyl in a final volume of 200 µL. The 96-well microtiter plate was incubated aerobically at 15°C until early exponential phase (OD_600_ = 0.3). Subsequently, the cultures were incubated under static conditions for 48 h to allow biofilm formation. The growth achieved was quantified by measuring the optical density at 600 nm after resuspending the content of each well using a pipette. For biofilm quantification, the content of the plate was discarded, and the attached bacteria were fixed with 200 µL of 99% methanol (Panreac). After fixation for 2 min, the methanol was discarded and crystal violet was added to each well and incubated for 5 min. The excess dye was rinsed off with distilled water, and the dye bound to the biofilm was solubilized with 200 µL of 33% (vol/vol) glacial acetic acid (Panreac). Biofilm quantification was performed by measuring absorbance at 570 nm in an iMark Microplate Reader (Bio-Rad). The results shown are the mean values of three independent experiments with four technical replicates each one. Statistical significance was determined by Student’s *t* test with a threshold *P* value < 0.05.

## RESULTS

### Purification of C- and N-terminal domains of PbtA

**FIG 2:**
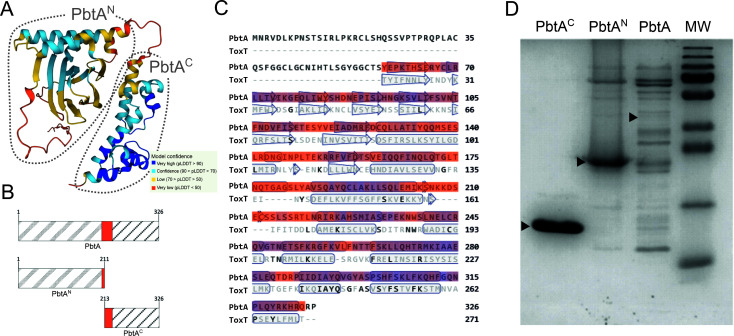
(**A**) AlphaFold protein model of PbtA (UniProtKB accession: A0A289GIJ0) where colors represent per-residue confidence score (pLDDT). (**B**) Schematic representation of the recombinant proteins produced in this work (PbtA, PbtA^C^, and PbtA^N^) where the numbers correspond to the amino acid positions in PbtA. (**C**) Amino acid alignment of PbtA and *V. cholerae* ToxT (UniProtKB accession: A5F384). (**D**) SDS-PAGE gel of PbtA, PbtA^C^, and PbtA^N^ after purification and concentration process. Black triangles denote the expected migration of each recombinant protein: PbtA (38 kDa), PbtA^C^ (14.25 kDa), and PbtA^N^ (24.5 kDa).

The transcriptional regulator PbtA is 326 amino acids in length and exhibits a two-domain arrangement commonly found in AraC-like transcriptional regulators such as ToxT (38, 39) (Fig. 2A). The N-terminal domain of PbtA (PbtA^N^) is predicted to comprise the first 210 amino acids. Following a short linker of ca 15 aa, the C-terminal domain of PbtA (PbtA^C^) (region S228-P326) contains the characteristic helix-turn-helix (HTH) DNA-binding domain. BLASTP search showed that ToxT of *V. cholerae* (UniProtKB accession: A5F384) is the closest homologue of PbtA whose function has been studied (38). The overall sequence identity shared between the two proteins is only 14.23%, although it increases to 29% (48% similarity) when the alignment is limited to their C-terminal domains (Fig. 2C). Thus, PbtA does not exhibit significant aa similarity to any previously characterized AraC-like regulators.

DNA sequences encoding full-length PbtA or PbtA^C^ and PbtA^N^ domains (Fig. 2B) were cloned into the expression vector pET20b(+) in frame with His-tags, and their expression was induced in *E. coli*. The resulting proteins were purified using Ni^2+^-affinity chromatography. PbtA^C^ (14.25 kDa) and PbtA^N^ (24.5 kDa) domains were successfully produced and purified (Fig. S1; Fig. 2D). However, the complete PbtA (38 kDa) protein was predominantly found in the insoluble fraction, suggesting low solubility. In agreement with this, the purification resulted in a low yield of the protein (Fig. S1), and several concentration attempts led to protein degradation (Fig. 2D). Given the challenges with solubility and protein stability, it was decided not to use the complete PbtA protein in subsequent experiments. Thus, the feasible production and purification of PbtA^C^ and PbtA^N^ allowed their utilization for *in vitro* studies.

### PbtA C-terminal domain binds directly to the *irp*-HPI promoters P*frpA* and P*frpC*

To assess the binding ability of the two domains of PbtA (PbtA^C^ and PbtA^N^) to 3′end biotin-labeled DNA probes, EMSA was employed. In the initial set of EMSAs, DNA probes of ca. 350 bp that encompassed the *irp*-HPI promoters P*pbtA*, P*frpA*, and P*frpC* (Fig. 1) were used. Specifically, P*frpA* probe corresponds to the *pbtB-frpA* intergenic region of 333 bp and controls the expression of piscibactin biosynthesis and transport genes (*frpAirp1-9*). P*frpC* controls the expression of *frpC* and *frpB* transport genes. P*pbtA* and P*frpC* probes include the 330- and 340-bp DNA region upstream of the ATG start codon of *pbtA* and *frpC*, respectively. PbtA does not regulate its own promoter (24) so the P*pbtA* probe was used as EMSA-negative control. EMSA assays were performed as described in experimental procedures, and the interpretation of the results relied on two indicators: (1) the decrease in the band intensity of the free DNA compared to the lane that contained only DNA, and (2) the appearance of a band at the top of the gel. These two indicators revealed the formation of the PbtA-DNA complex without relying on a conventional shift in DNA gel mobility, which was not possible to observe due to the high isoelectric point (pI) of the PbtA^C^ (pI = 10.43).

**FIG 3:**
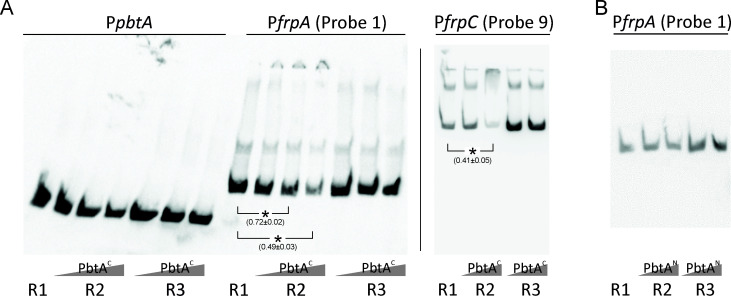
Electrophoretic mobility shift assays of PbtA^C^ (**A**) and PbtA^N^ (**B**) binding to probes encompassing *irp*-HPI promoters (P*pbtA*, P*frpA,* and P*frpC*). R1 denotes the labeled probe controls and does not include protein in the reaction; R2 includes increasing concentration of PbtA^C^ or PbtA^N^; R3 denotes reaction controls that include protein and an excess of non-labeled DNA. Asterisks denote statistical significance (*P < 0.05), and the relative quantification of the band is denoted within the parentheses.

**FIG 4:**
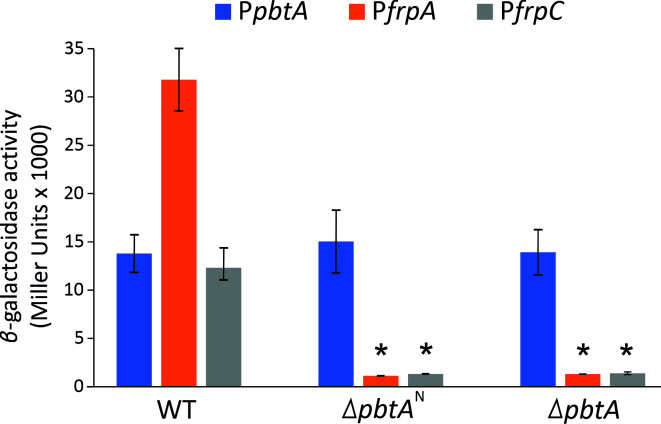
Transcriptional activity of the *irp*-HPI promoters P*pbtA*, P*frpA,* and P*frpC* in either *V. anguillarum* RV22 wild type strain (WT), used as the parental strain, or its derivative Δ*pbtA*^N^ and Δ*pbtA* defective mutants. Asterisks denote statistical significance, *P < 0.05.

The results of the EMSA assays showed that PbtA^C^ caused a reduction in the relative band intensity, denoting mobility shift, in the DNA probes that cover P*frpA* and P*frpC* promoter sequences. P*frpA* and P*frpC* showed relative quantifications of 0.49 ± 0.03 and 0.41 ± 0.03, respectively, when 1 pmol of PbtA^C^ was added to the EMSA reaction (Fig. 3A). As expected, it did not bind to its own promoter (P*pbtA*) (Fig. 3A). These results indicate that PbtA binds directly to piscibactin promoters P*frpA* and P*frpC*. In addition, the PbtA^N^ was unable to bind any of the DNA probes tested (Fig. 3B). This finding suggests that there are no DNA-binding determinants present in the N-terminal domain of the protein and that PbtA can bind to the target DNA sequences as a monomer. Interestingly, deletion of PbtA^N^ leads to a significant reduction in P*frpA* and P*frpC* transcriptional activity, matching the expression levels observed in the PbtA null mutant (Fig. 4). This result clearly suggests that the N-terminal domain of PbtA is required for its functionality and consequently for the expression of *irp* genes.

**FIG 5:**
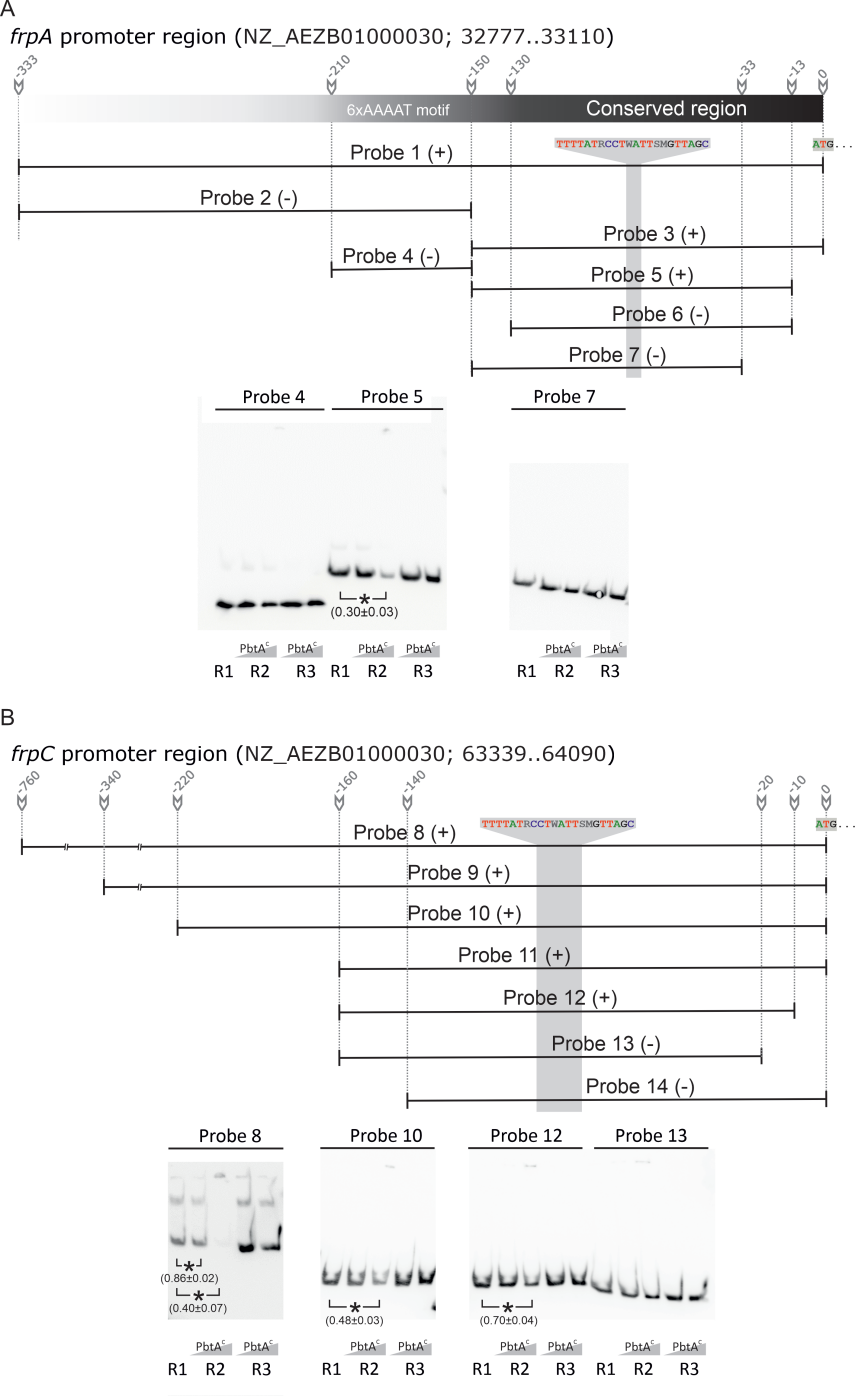
Schematic representation of *frpA* (**A**) and *frpC* (**B**) promoter regions, probes used for EMSA, and representative results. Numbers correspond to nucleotides position counting from the ATG star codons. Two regions are depicted in the *frpA* promoter region according to their presence among *Vibrionaceae* versions of *irp*-HPI: one harboring a 6xAAAAT motif and the other containing a conserved region. R1 denotes the labeled probe controls and does not include protein in the reaction; R2 includes increasing concentration of PbtA^C^; R3 denotes reaction controls that include protein and an excess of non-labeled DNA. Asterisks denote statistical significance (* *P* < 0.05), and the relative quantification of the band is denoted within the parentheses.

**FIG 6:**
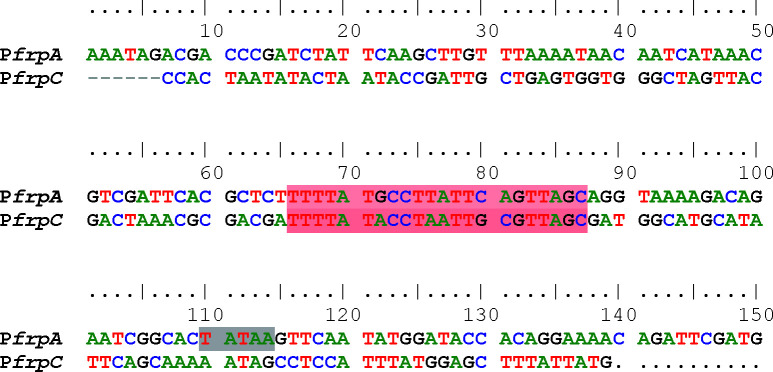
Alignment of ca. 150 bp of P*frpA* and P*frpC* regions. A conserved motif is highlighted in red. Gray shade denotes probable −10 promoter motif.

Further EMSA assays were performed to identify specific region(s) within the piscibactin promoters P*frpA* and P*frpC* that PbtA binds to (Fig. 5). These assays aimed to narrow down the PbtA binding region. The *pbtB-frpA* intergenic region with a size of approximately 333 bp includes a conserved region among all variants of *irp*-HPI island proximal to the ATG of *pbtA*, and a low complexity sequence with six repeats of an AAAAT motif (between positions 137 and 167) (24) (Fig. 5A). Upon testing the low complexity region by EMSA, the formation of a PbtA-DNA complex was not detected (Probe 4 in Fig. 5A). The minimum DNA fragment of P*frpA* required for a positive EMSA result was the region spanning from −13 to −150 of the ATG start codon (Probe 5 in Fig. 5A). In P*frpC,* the minimum EMSA-positive region was defined as that spanning from positions −10 to −160 (Probe 12 in Fig. 5B). Interestingly, the PbtA^C^ showed a higher affinity for the probes encompassing the larger *frpC* promoter regions (e.g., relative quantification of Probe 8 was 0.40 ± 0.07) than the smaller ones: Probe 10 (0.48 ± 0.03) and Probe 12 (0.70 ± 0.04) (Fig. 5B). We hypothesized that PbtA could have the property to bind to multiple binding sites in relatively large and diffuse DNA regions. Additionally, the EMSA-positive probes of P*frpA* and P*frpC* promoters would contain the −10 and −35 promoter elements and share a sequence of 22 bp with the motif 5′-TTTTATRCCTWATTSMGTTAGC-3′ (Fig. 6). It is interesting to note that both *irp*-HPI promoter regions, P*frpA* and P*frpC,* have an extremely low G + C content (Fig. 1). This suggests that a low G + C content may be a requirement for PbtA binding to DNA.

### The inactivation of *pbtA* induces changes in the whole-genome expression profile

To gain insight into the putative role of PbtA in the modulation of *V. anguillarum* whole-genome expression, an RNAseq assay was conducted. Therefore, the transcriptomic profile of the RV22 wild-type strain was compared to that of a *pbtA* null mutant (RV22 Δ*pbtA*). Both strains were subjected to growth at low temperature (15°C) under weak iron-restrictive conditions, to mimic the iron-starved conditions encountered during the host–pathogen interaction and to ensure the expression of virulence factors (17).

**FIG 7:**
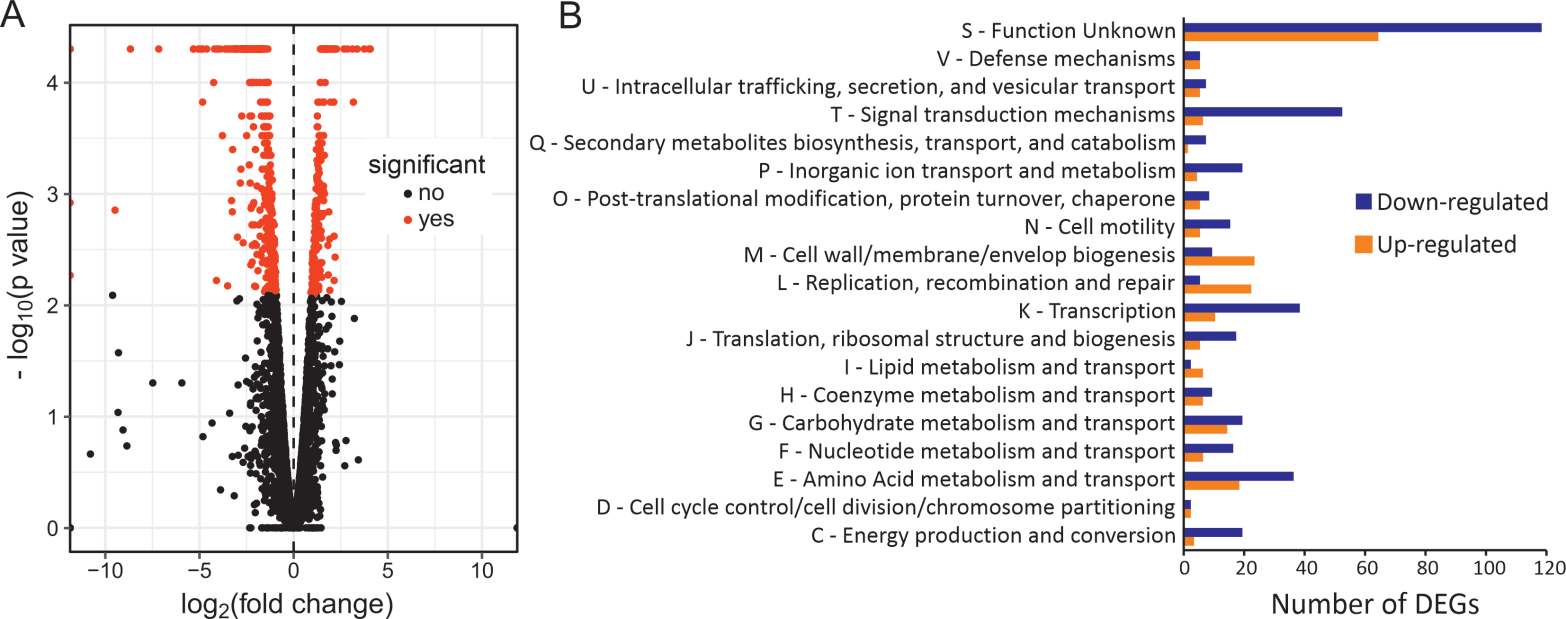
Modifications in *V. anguillarum* transcriptome after *pbtA* inactivation (expression values of Δ*pbtA* mutant compared to the wild-type strain). (**A**) Volcano plot showing differentially expressed genes (DEGs). (**B**) Functional classification of DEGs into KEGG categories.

The transcriptomic analysis showed that approximately 16% of *V. anguillarum* RV22 genome, specifically 569 out of 3,621 genes, were differentially expressed: 370 genes were downregulated, whereas 199 genes were upregulated upon PbtA deletion (Fig. 7A) (Table S4). The differentially expressed genes (DEGs) were grouped into functional Kyoto Encyclopedia of Genes and Genomes (KEGG) categories (Fig. 7B). The results indicate that the deletion of *pbtA* results in a downregulation of genes related to signal transduction mechanisms (T), transcription (K), amino acid metabolism and transport (E), energy production and conversion (C), inorganic ion transport and metabolism (*P*), and translation, ribosomal structure and biogenesis (J). Conversely, functions related to replication, recombination, and repair (L) and cell wall/membrane biogenesis (M) were upregulated. Numerous proteins (ca. 180) with unknown function were found within the differentially expressed genes. Differential expression of the down-expressed genes *frpA*, *vanT*, and *hcp1*, as well as the up-expressed *wza*, was verified by RT-qPCR, showing 2^(-ΔΔct)^ values of 0.07 ± 0.02 (mean ± SD), 0.73 ± 0.18, 0.37 ± 0.18, and 1.95 ± 0.51, respectively. These results strongly suggest that PbtA exerts, directly or indirectly, a global regulatory effect in *V. anguillarum* transcriptome.

**FIG 8:**
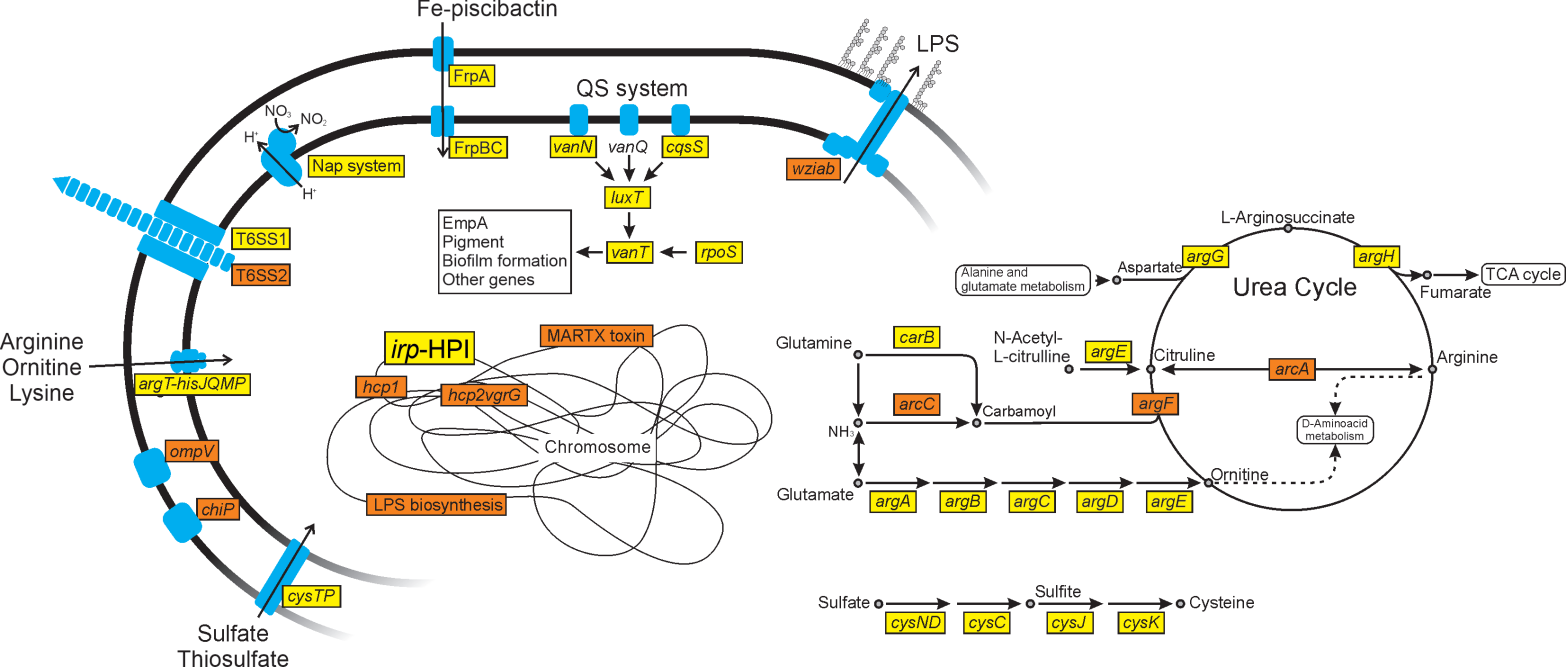
Schematic representation showing the relevant differentially expressed genes (DEGs) (Δ*pbtA* mutant compared to the wild-type strain) related to virulence, and arginine and sulfafe metabolisms. Upregulated genes after inactivation of PbtA are marked in orange, whereas downregulated genes are shown in yellow.

The most representative DEGs related to metabolism and virulence are illustrated in Fig. 8. Specifically, the expression of genes *napFDABC* encoding periplasmic nitrate reductase (Nap system) decreased by 3.8-fold. Genes related to sulfur metabolism, including *cysKDNCGJ*, exhibit an overall downregulation of 2.7-fold. Additionally, genes involved in sulfate uptake, such as *cysT* and *cysP*, showed a 3.9-fold downregulation. The inactivation of PbtA also impacts arginine biosynthesis and transport. The expression of genes involved in the linear synthesis pathway of arginine, *argA-H* genes (40), was diminished by 5.1-fold. The arginine transport system encoded by the gene cluster *argT-hisJQMP* showed a significant 2.8-fold decrease in expression. In agreement with this, the pathway involved in *de novo* synthesis of arginine as well as its import from the extracellular environment showed a reduced expression. Complementary, the alternative pathway for arginine synthesis was 14.8-fold induced. This pathway involves three enzymatic steps mediated by ArgF and ArcAC (40). The gene *arcA*, which encodes an arginine deiminase responsible for the interconversion of citrulline and arginine, showed a significant 16.8-fold upregulation.

When examining the list of differentially expressed genes, several well-known virulence factors were found (Table 1). As previously described, PbtA is required for the expression of the piscibactin system (24). Congruently, *irp*-HPI genes were found within the most downregulated differentially expressed genes (−17.4-fold). A slight induction of vanchrobactin genes was also observed, with an overall fold change of 1.4. However, significant changes were only detected in vanchrobactin export (*vabS*), utilization (*vabH*), and regulatory (*vabR*) genes. The vanchrobactin TonB-dependent outer membrane transporter gene *fvtA* was constitutively expressed under both conditions. The overall expression of the MARTX toxin, encoded by the *rtxACHBDE* operon (41), showed an increase of 2.1-fold. Among these genes, the toxin acyltransferase *rtxC* and the toxin ABC transporters component *rtxE* were significantly upregulated, with a 3.4-fold and 2.0-fold increase, respectively. More notably, genes related to the T6SS1 showed a 6.4-fold downregulation, whereas the T6SS2 was 2.9-fold induced. In addition, several T6SS-related genes located elsewhere in *V. anguillarum* genome were also differentially expressed. Specifically, the effector proteins and components of the structural puncturing device *hcp1*, *hcp2,* and *vgrG* showed a reduction in their expression of 143.5-, 39.7-, and 5.72-fold, respectively. Two copies of the structural component PAAR domain displayed a divergent expression, with one showing a 4.6-fold induction and the other a 2.64-fold repression. Deletion of PbtA appears to also have an impact on the outer membrane components of *V. anguillarum*. Specifically, the expression of two major porins, OmpV and ChiP, was induced 3.4-fold and 2.3-fold, respectively. The genes involved in lipopolysaccharide (LPS) biosynthesis showed a 2.6-fold upregulation, indicating an increase in LPS production. Moreover, the genes responsible for exopolysaccharide transport and assembly, *wzbai* and *wbfcD* operons, were twofold upregulated in the mutant strain. These findings suggest that the deletion of PbtA leads to a remodulation of *V. anguillarum* outer membrane, including an increased expression of some porins and LPS.

**TABLE 1:** Most relevant differentially expressed genes related to metabolic and virulence functions after deletion of *pbtA* in *V. anguillarum^a^*

	Expression level (FPKM)		Fold change
	Wild-type	Δ*pbtA*	
**Metabolism**			
**Nitrate reductase**			
*narQ* (WP_019281627.1)	160.8	64.3	−2.5
*napFABCD*	15.0	3.9	−3.8
**Sulfur metabolism**			
*cysKDNCGJ*	297.3	109.2	−2.7
*cysTP*	140.2	36.2	−3.9
**Arginine metabolism**			
*argABCEFGH*	2121.2	418.8	−5.1
*carAB*	370.7	62.7	−5.9
*arcAC argF*	9.1	134.7	14.8
*argT hisMQP*	352.1	124.5	−2.8
**Virulence factors**			
**Piscibactin**			
*pbtAB irp1234589 frpBC*	213.6	12.2	−17.6
*frpA*	428.2	26.0	−16.4
**Vanchrobactin**			
*vabABCDEFGHSR mbtH*	964.6	1374.8	1.4
*fvtA*	2015.7	1978.0	ns
**MARTX**			
*rtxACHBDE*	1572.7	3379.9	2.1
*rtxA* (WP_019282576.1)	1396.5	HIDATA	nd
*rtxC* (WP_010848330.1)	956.6	3222.9	3.4
*rtxH* (WP_013856638.1)	6553.1	12733.3	ns
*rtxB* (WP_019282577.1)	174.3	326.0	ns
*rtxD* (WP_010319619.1)	264.1	431.2	ns
*rtxE* (WP_017047016.1)	91.4	186.3	2.0
**Type VI secretion system**			
T6SS1	193.4	30.2	−6.4
T6SS2	510.4	1469.9	2.9
PARR domain (WP_019283293.1)	14.1	64.7	4.6
PARR domain (WP_010318209.1)	144.3	54.6	−2.6
*hcp1* (WP_013868180.1)	1373.2	9.6	−143.5
*hcp2* (WP_013857791.1)	295.5	7.4	−39.7
*vgrG*	51.1	8.9	−5.7
**Porins**			
*ompV* (WP_019282463.1)	294.5	1005.3	3.4
*chiP* (WP_019282602.1)	57.2	132.1	2.3
**Lipopolysaccharide/ Exopolysaccharide**			
Biosynthesis	86.6	221.2	2.6
Transport and assemble	58.5	116.5	2.0
**Quorum sensing**			
*rpoS* (WP_017047531.1)	1128.9	144.4	−7.8
*luxT* (WP_013867986.1)	432.4	201.3	−2.1
*vanN* (WP_019281872.1)	64.3	27.8	−2.3
*vanT* (WP_017043694.1)	579.6	66.2	−8.8
*cqsS*	93.3	11.8	−7.9

^*a*^ Fold change values with *P* < 0.05 are shown; ns, non-significant differences detected; nd, non-determined; HIDATA, too many fragments in locus.

**FIG 9:**
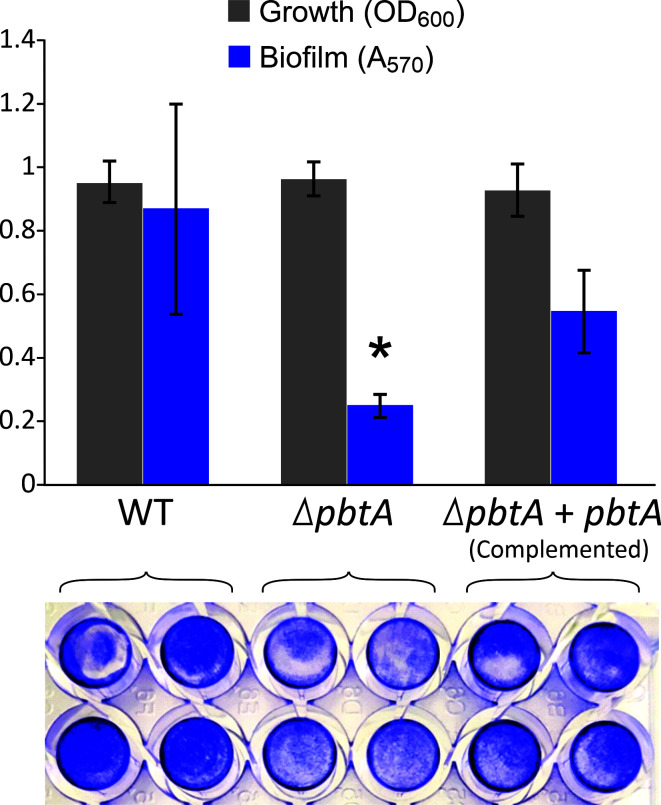
Biofilm formation of *V. anguillarum* wild-type, its derivative *pbtA* defective mutant, and complemented strain. Biofilm quantification was performed using the crystal violet assay. The results shown are the mean ± SD of three independent experiments with four technical replicas. Asterisk denotes statistically significant differences. Representative image of a biofilm assay is shown.

As mentioned earlier, numerous genes encoding functions grouped in the categories signal transduction mechanisms (T) and transcription (K) were differentially expressed. These include ca. 70 genes encoding some diguanylate cyclases, LysR- and AraC-type transcriptional regulators (Table S4). The expression of *rpoS*, the RNA polymerase sigma factor S (42), showed a significant 7.8-fold decrease (Table 1). Notably, the inactivation of PbtA led to a significant downregulation of components of the quorum sensing system, including the master regulator VanT (8.8-fold decrease). The sensor kinase proteins, VanN and CqsS, responsible for signal recognition of autoinducers AI-1 and CAI-1 and the TetR-like transcriptional regulator LuxT showed a 2.3-, 7.9-, and 2.1-fold downregulation. Quorum sensing plays a central role in bacteria regulation and virulence, including biofilm formation (43). Congruently, a reduced capacity for biofilm formation was verified in the *pbtA* defective mutant compared to wild-type strain (Fig. 9). This finding raises the question of whether the observed changes in the whole-genome expression pattern associated with PbtA result from the direct interaction of PbtA with the promoters of regulated genes, or if additional players are involved.

### PbtA would act as both activator and repressor in the modulation of genes located outside of the *irp*-HPI genomic island

**FIG 10:**
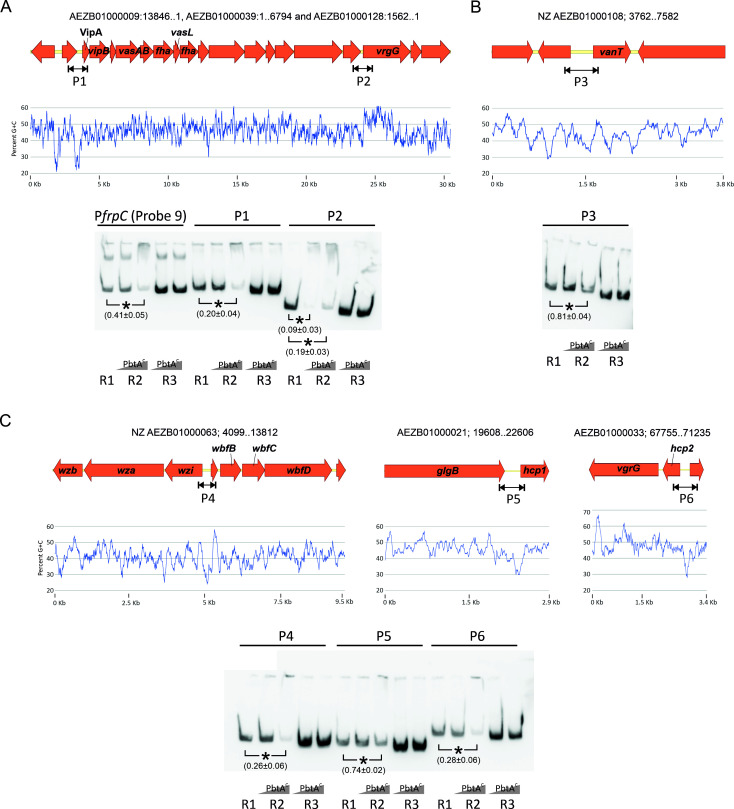
Electrophoretic mobility shift assays of *vipA, vgrG, vanT, wziab, hcp1,* and *hcp2* promoter regions. P*frpC* (probe 9) was used as control. Gene cluster organization and GC content are shown for each genomic region. EMSAs were performed as described in Material and Methods: R1 denotes the labeled probe controls and does not include PbtA^C^ protein in the reaction; R2 includes increasing concentration of PbtA^C^; R3 denotes reaction controls that include protein and an excess of non-labeled DNA. Asterisks denote statistical significance (*P < 0.05), and the relative quantification of the band is denoted within the parentheses.

We have shown above that PbtA efficiently binds to *irp*-HPI promoter regions, but its ability to directly modulate differentially expressed genes (DEGs) located outside of the genomic island was not elucidated. Thus, EMSA assays were performed to study whether PbtA directly binds to promoters of certain virulence-related genes. The promoter regions tested included those of the downregulated genes *vanT*, T6SS genes *vipA*, *hcp1*, *hcp2,* and *vgrG*, as well as the promoter of LPS transport and assembly genes *wziab*. LPS genes were over-expressed after inactivation of PbtA (see above). DNA probes and EMSA results are shown in Fig. 10. The results showed that PbtA^C^ binds to the promoters of *vipA* (0.20 ± 0.04) (Fig. 10A), *hcp2* (0.28 ± 0.06), and LPS (*wzi*) (0.26 ± 0.06) (Fig. 10C) with higher affinity than *irp* promoters P*frpA* (0.49 ± 0.03) and P*frpC* (0.41 ± 0.05) (Fig. 3A and 10A). It exhibited a higher binding affinity to the promoter region of *vgrG* even at the lowest protein concentration tested (0.09 ± 0.03) (Fig. 10A) and was also capable of binding to the promoter region of *vanT* (0.81 ± 0.04) (Fig. 10B) and *hcp1* (0.74 ± 0.02) (Fig. 10C), albeit with lower affinity. These findings greatly suggest that PbtA may play a direct role in regulating the expression of a great range of genes located elsewhere in *V. anguillarum* genome, including important virulence factors described in *Vibrio* species. Interestingly, PbtA efficiently binds to promoters of both down- and up-expressed genes, which suggests that PbtA would act as activator or repressor, depending on the target promoter.

## DISCUSSION

AraC-like proteins involved in the regulation of carbon metabolism (e.g., the arabinose operon regulator [AraC] ), are active as dimers, and their activation is induced by effector molecules that bind to the N-terminal domain (44). In contrast, those involved in stress response (e.g., SoxS, MarA, and Rob) and regulation of virulence factors (e.g., ToxT) can function as monomers (45–48). EMSA results provided clear evidence that the C-terminal domain of PbtA functions by directly binding to the promoters of the piscibactin biosynthesis and transport genes (P*frpA* and P*frpC*). The N-terminal domain is not needed for stable DNA binding of PbtA *in vitro*, but it is required for its functionality. Thus, it is hypothesized that the N-terminal domain of PbtA has a regulatory role, potentially functioning as a sensor for environmental signals or effector molecules and mediating a probable dimerization of PbtA to orchestrate its regulatory effects. AraC transcriptional factors involved in regulation of virulence factors usually mediate regulatory responses to a wide variety of environmental signals such as temperature, pH, oxidative stress, salinity, etc. (48–50). Further studies are necessary to determine the specific effector(s) or environmental signal(s) that trigger the response of PbtA and to identify its mode of action.

More notably, the results demonstrate that PbtA not only is required for the expression of the *irp*-HPI genes but also modulates approximately 16% of *V. anguillarum* transcriptome. This includes the ability to produce piscibactin (encoded by *irp*-HPI) and the induction of genes related to nitrate, arginine, and sulfate metabolism, Type VI Secretion System 1 (T6SS1), and quorum sensing. Simultaneously, it leads to the repression of genes associated with lipopolysaccharide (LPS) production, Type VI Secretion System 2 (T6SS2), MARTX toxin, and major porins like OmpV and ChiP. Repression of outer membrane components such as LPS would allow the pathogen to evade the host immune system response and persist within the host (51). Interestingly, most of these factors, including T6SS, were found to be essential for *V. anguillarum* persistence during the bacteria–host interaction (12). The contrasting effect of PbtA on the expression of each T6SS system is remarkable, as it activates T6SS1 and represses T6SS2. The expression pattern and the role in fitness and virulence of each T6SS varies among *Vibrio* species and also depends on the specific subset of effector proteins (52, 53). The concrete role of T6SSs in *V. anguillarum* needs to be further studied. Overall, the results show that PbtA modulates the expression of numerous factors known to be required for virulence in Gram-negative bacteria (41, 54–56). RNAseq results suggest that PbtA primarily functions as a transcriptional activator, as most differentially expressed genes showed reduced expression after its inactivation. Specifically, we proved that PbtA directly interacts with the promoter region of down-expressed genes such as the quorum sensing master regulator *vanT*, and structural components of the T6SS1 (*vipA*) and its probable puncturing device (*hcp1*, *hcp2,* and *vgrG* genes). But it also binds to the promoter region of LPS transport and assemble genes (*wziab*), whose expression significantly increases in the PbtA mutant strain. This result suggests that PbtA may also act as a repressor. Although most characterized members of this family act as transcriptional activators, a few have been found to function exclusively or additionally as repressors (39). In this concern, ToxT requires dimerization for the activation of most target genes, such as cholera toxin gene *ctxA*, but it can also function as a monomer to activate other genes (57, 58). In addition, it binds as a monomer to three “toxbox” sequences within the *msh* operon to inhibit *mshA* transcription (59, 60). Our results altogether provide strong evidence that PbtA is directly responsible for modulating the transcription of genes located elsewhere in *V. anguillarum* genome and that its role as activator or repressor could depend on the specific target gene.

Due to the diverse mechanisms of action exhibited by AraC-like regulators, it can be challenging to accurately predict the binding sites and the genes they modulate (39). Several members of the family interact with the RNAP α-subunit C-terminal domain (α-CTD) for transcription activation (45, 61, 62). We have demonstrated that PbtA efficiently binds to DNA regions of approximately 150 base pairs, which share a 22-bp sequence with the motif 5′-TTTTATRCCTWATTSMGTTAGC-3′, and extend from position −10 of the ATG start codons of both *frpA* and *frpC* promoters. Thus, we hypothesized that the presence of this sequence motif would be an important feature for PbtA binding to these regions. However, PbtA exhibited a high affinity for the promoter region of *vgrG* and efficiently binds to the promoters of *hcp2* and LPS production (*wzi* promoter region), and these promoter regions do not share the DNA motif identified in the *irp*-HPI. Apparently, the unique common characteristic shared by the promoter regions regulated by PbtA is a low GC content. AraC-like regulators commonly recognize specific nucleotide motifs or structural DNA features, such as the intrinsically curved A + T rich DNA sequences, which are often located in or near the −30 region of transcription start sites (45). Thus, they can play a role in counteracting the gene silencing effects mediated by global negative H-NS regulators, one of the most studied xenogeneic silencers (8, 63). A noteworthy feature of the AraC/XylS family is the capacity of certain members (e.g., AraC or MelR) to mediate DNA looping, forming the so-called “repression loops” (44, 64). Further studies will be needed to investigate the functional significance of PbtA preference for AT-rich promoter regions and to elucidate the precise mechanisms by which PbtA recognizes and interacts with its target DNA sequences.

*V. anguillarum* possess two quorum sensing systems: an acyl-homoserin lactone (AHL) system involving the pair VanI/VanR, and a three-channel system that modulates the expression of the target genes through VanT (65). Our results show that PbtA plays a role in modulating the quorum sensing system in *V. anguillarum*. The ability of PbtA for binding *vanT* promoter region suggests that this effect could be a direct interaction. Some virulence factors of *V. anguillarum* such as EmpA metalloprotease, pigment production, and biofilm formation are regulated by quorum sensing (65–67). In addition, RpoS of *V. anguillarum* plays important roles in bacterial adaptation to environmental stresses and pathogenicity, promoting the expression of quorum sensing and virulence factors (42, 68). However, the role of quorum sensing in *V. anguillarum* virulence remains unclear since some studies suggest that it would not play a major role (65, 66, 69). Interestingly, recent research has revealed that the importance of quorum sensing in virulence varies greatly among *V. anguillarum* strains (70). By influencing the quorum sensing system, PbtA may have far-reaching effects on the overall gene expression patterns and behaviors of *V. anguillarum*.

Besides virulence factors themselves, the inactivation of PbtA also leads to the downregulation of various cellular processes that may play roles in the bacteria–host interaction. These include the periplasmic nitrate reductase, Nap operon, which act as an alternative electron acceptor in the absence of O_2_ (71), or sulfate metabolism (72). The genes encoding sulfate metabolism are repressed by cysteine, the end-product of the pathway (73, 74). Given that cysteine is a precursor for piscibactin synthesis (75), the non-production of the siderophore likely results in an intracellular accumulation of this amino acid, contributing to the observed downregulation of sulfate metabolism. The results suggest that the deletion of *pbtA* likely abolishes the *de novo* arginine synthesis and also its import from the extracellular environment. Bacterial arginine requirements would be met by the upregulation of *arcA*, an arginine deiminase required for the alternative pathway for arginine synthesis (76). Arginine importance is not restricted to protein synthesis, as this amino acid can be used as nitrogen and carbon source and, along with its precursor ornithine, is a substrate for the synthesis of polyamines as well as proline. Additionally, the involvement of arginine pathway in acid resistance under anaerobic conditions in *E. coli* (40) raises the possibility that this pathway may also play a role in *V. anguillarum* persistence within the host.

Previous results showed that certain *V. anguillarum* strains exhibit a remarkable host range plasticity (14, 15), which was associated with the acquisition of the *irp*-HPI genomic island through horizontal gene transfer (16, 17). The present work shows that this adaptive capability is likely achieved through comprehensive changes in the transcriptome of the bacteria and that these changes are mediated by the master regulator PbtA. The global regulatory effect of PbtA on *V. anguillarum* transcriptome counterbalances the reduced growth ability of this pathogen at cold temperatures (17) by inducing the overproduction of numerous virulence traits and promoting evasion of host immunity. Our results reinforce the idea that the acquisition of a genomic island can directly mediate changes in the expression of the core genome, as it was suggested for the pathogenicity island SPI-1 of *Salmonella* or PAPI-1 of *Pseudomonas aeruginosa* (77, 78).

Overall, our results provide valuable insight into the significant role of PbtA, and hence about *irp*-HPI acquisition, in modulating the expression of a plethora of *V. anguillarum* core genome genes, virulence factors, and behavior of *V. anguillarum*, with notable implications in adaptation to the host and pathogenesis. Further research will unravel the complete role of PbtA as a master regulator in modulating gene expression in *Vibrionaceae*. Indeed, the present work clearly shows that horizontally acquired DNA has the capacity to remodel the bacterial transcriptome.

## SUPPLEMENTAL MATERIAL

Supplemental tables and figure msphere.00596-23-s0001.docx Tables S1 to S3 and Figure S1.

Table S4 msphere.00596-23-s0002.xlsx Expression data set.

ASM does not own the copyrights to Supplemental Material that may be linked to, or accessed through, an article. The authors have granted ASM a non-exclusive, world-wide license to publish the Supplemental Material files. Please contact the corresponding author directly for reuse.

## References

[1] Dobrindt U, Hochhut B, Hentschel U, Hacker J. 2004. Genomic islands in pathogenic and environmental microorganisms. Nat Rev Microbiol 2:414–424. doi:10.1038/nrmicro884 15100694

[2] Hazen TH, Pan L, Gu J-D, Sobecky PA. 2010. The contribution of mobile genetic elements to the evolution and ecology of vibrios. FEMS Microbiol Ecol 74:485–499. doi:10.1111/j.1574-6941.2010.00937.x 20662928

[3] Juhas M, van der Meer JR, Gaillard M, Harding RM, Hood DW, Crook DW. 2009. Genomic Islands: tools of bacterial horizontal gene transfer and evolution. FEMS Microbiol Rev 33:376–393. doi:10.1111/j.1574-6976.2008.00136.x 19178566 PMC2704930

[4] Bruto M, James A, Petton B, Labreuche Y, Chenivesse S, Alunno-Bruscia M, Polz MF, Le Roux F. 2017. Vibrio crassostreae, a benign oyster colonizer turned into a pathogen after plasmid acquisition. ISME J 11:1043–1052. doi:10.1038/ismej.2016.162 27922600 PMC5364345

[5] Safa A, Jime JS, Shahel F. 2020. Cholera toxin phage: structural and functional diversity between Vibrio cholerae biotypes. AIMS Microbiol 6:144–151. doi:10.3934/microbiol.2020009 32617446 PMC7326730

[6] Park C, Zhang J. 2012. High expression hampers horizontal gene transfer. Genome Biol Evol 4:523–532. doi:10.1093/gbe/evs030 22436996 PMC3342876

[7] Duan B, Ding P, Navarre WW, Liu J, Xia B. 2021. Xenogeneic silencing and bacterial genome evolution: mechanisms for DNA recognition imply multifaceted roles of xenogeneic silencers. Mol Biol Evol 38:4135–4148. doi:10.1093/molbev/msab136 34003286 PMC8476142

[8] Stoebel DM, Free A, Dorman CJ. 2008. Anti-silencing: overcoming H-NS-mediated repression of transcription in gram-negative enteric bacteria. Microbiology (Reading) 154:2533–2545. doi:10.1099/mic.0.2008/020693-0 18757787

[9] Le Roux F, Blokesch M. 2018. Eco-evolutionary dynamics linked to horizontal gene transfer in vibrios. Annu Rev Microbiol 72:89–110. doi:10.1146/annurev-micro-090817-062148 29897833

[10] Toranzo AE, Magariños B, Avendaño-Herrera R. 2017. Vibriosis: *Vibrio anguillarum*, *V. ordalii* and *Aliivibrio salmonicida*, p 314–333. In Fish viruses and bacteria: Pathobiology and protection. CABI, Wallingford.

[11] Hickey ME, Lee JL. 2017. A comprehensive review of Vibrio (Listonella) anguillarum: ecology, pathology and prevention. Rev Aquac 1893:1–26.

[12] Guanhua Y, Wang C, Wang X, Ma R, Zheng H, Liu Q, Zhang Y, Ma Y, Wang Q. 2018. Complete genome sequence of the marine fish pathogen Vibrio anguillarum and genome-wide transposon mutagenesis analysis of genes essential for In vivo infection. Microbiol Res 216:97–107. doi:10.1016/j.micres.2018.08.011 30269861

[13] Lemos ML, Balado M. 2020. Iron uptake mechanisms as key virulence factors in bacterial fish pathogens. J Appl Microbiol 129:104–115. doi:10.1111/jam.14595 31994331

[14] Castillo D, Alvise PD, Xu R, Zhang F, Middelboe M, Gram L, Zhaxybayeva O. 2017. Comparative genome analyses of Vibrio anguillarum strains reveal a link with pathogenicity traits. mSystems 2:e00001-17. doi:10.1128/mSystems.00001-17 28293680 PMC5347184

[15] Rønneseth A, Castillo D, D’Alvise P, Tønnesen Ø, Haugland G, Grotkjaer T, Engell-Sørensen K, Nørremark L, Bergh Ø, Wergeland HI, Gram L. 2017. Comparative assessment of Vibrio virulence in marine fish larvae. J Fish Dis 40:1373–1385. doi:10.1111/jfd.12612 28160295

[16] Balado M, Lages MA, Fuentes-Monteverde JC, Martínez-Matamoros D, Rodríguez J, Jiménez C, Lemos ML. 2018. The siderophore piscibactin is a relevant virulence factor for Vibrio anguillarum favored at low temperatures. Front Microbiol 9:1766. doi:10.3389/fmicb.2018.01766 30116232 PMC6083037

[17] Lages MA, Balado M, Lemos ML. 2019. The expression of virulence factors in Vibrio anguillarum is dually regulated by iron levels and temperature. Front Microbiol 10:2335. doi:10.3389/fmicb.2019.02335 31681201 PMC6803810

[18] Osorio CR, Juiz-Río S, Lemos ML. 2006. A siderophore biosynthesis gene cluster from the fish pathogen Photobacterium damselae subsp. piscicida is structurally and functionally related to the Yersinia high-pathogenicity Island. Microbiology (Reading) 152:3327–3341. doi:10.1099/mic.0.29190-0 17074903

[19] Lages MA, de la Fuente MC, Ageitos L, Martínez-Matamoros D, Rodríguez J, Balado M, Jiménez C, Lemos ML. 2022. Frpa is the outer membrane piscibactin transporter in Vibrio anguillarum: structural elements in synthetic piscibactin analogues required for transport. J Biol Inorg Chem 27:133–142. doi:10.1007/s00775-021-01916-1 34792655 PMC8840927

[20] Osorio C.R, Rivas AJ, Balado M, Fuentes-Monteverde JC, Rodríguez J, Jiménez C, Lemos ML, Waldor MK. 2015. A transmissible plasmid-borne pathogenicity island encodes piscibactin biosynthesis in the fish pathogen Photobacterium damselae subsp. piscicida. Appl Environ Microbiol 81:5867–5879. doi:10.1128/AEM.01580-15 26092457 PMC4551267

[21] Ruiz P, Balado M, Fuentes-Monteverde JC, Toranzo AE, Rodríguez J, Jiménez C, Avendaño-Herrera R, Lemos ML. 2019. The fish pathogen Vibrio ordalii under iron deprivation produces the siderophore piscibactin. Microorganisms 7:313. doi:10.3390/microorganisms7090313 31484388 PMC6780188

[22] Galvis F, Ageitos L, Rodríguez J, Jiménez C, Barja JL, Lemos ML, Balado M. 2021. Vibrio neptunius produces piscibactin and amphibactin and both siderophores contribute significantly to virulence for clams. Front Cell Infect Microbiol 11:750567. doi:10.3389/fcimb.2021.750567 34760718 PMC8573110

[23] Thode SK, Rojek E, Kozlowski M, Ahmad R, Haugen P. 2018. Distribution of siderophore gene systems on a Vibrionaceae phylogeny: database searches, phylogenetic analyses and evolutionary perspectives. PLoS One 13:e0191860. doi:10.1371/journal.pone.0191860 29444108 PMC5812596

[24] Lages MA, Lemos ML, Balado M. 2021. The temperature-dependent expression of the high-pathogenicity island encoding piscibactin in Vibrionaceae results from the combined effect of the AraC-like transcriptional activator PbtA and regulatory factors from the recipient genome. Front Microbiol 12:748147. doi:10.3389/fmicb.2021.748147 34867865 PMC8639528

[25] Lages MA, Ageitos L, Rodríguez J, Jiménez C, Lemos ML, Balado M. 2022. Identification of key functions required for production and utilization of the siderophore piscibactin encoded by the high-pathogenicity island irp-HPI in Vibrionaceae Int J Mol Sci 23:8865. doi:10.3390/ijms23168865 36012135 PMC9408133

[26] Lemos ML, Salinas P, Toranzo AE, Barja JL, Crosa JH. 1988. Chromosome-mediated iron uptake system in pathogenic strains of Vibrio anguillarum. J Bacteriol 170:1920–1925. doi:10.1128/jb.170.4.1920-1925.1988 2965144 PMC211051

[27] UniProt Consortium. 2021. UniProt: the universal protein knowledgebase in 2021. Nucleic Acids Res 49:D480–D489. doi:10.1093/nar/gkaa1100 33237286 PMC7778908

[28] Wolfsberg TG, Madden TL. 2001. Sequence similarity searching using the BLAST family of programs, . In Curr protoc protein SCI chapter 2:Unit2.5 10.1002/0471140864.ps0205s15 18429153

[29] Wilkins MR, Gasteiger E, Bairoch A, Sanchez JC, Williams KL, Appel RD, Hochstrasser DF. 1999. Protein identification and analysis tools in the ExPASy server. Methods Mol Biol 112:531–552. doi:10.1385/1-59259-584-7:531 10027275

[30] Wang RF, Kushner SR. 1991. Construction of versatile low-copy-number vectors for cloning, sequencing and gene expression in Escherichia coli. Gene 100:195–199. doi:10.1016/0378-1119(91)90366-J 2055470

[31] Mouriño S, Osorio CR, Lemos ML. 2004. Characterization of heme uptake cluster genes in the fish pathogen Vibrio anguillarum. J Bacteriol 186:6159–6167. doi:10.1128/JB.186.18.6159-6167.2004 15342586 PMC515166

[32] Martínez-García E, Goñi-Moreno A, Bartley B, McLaughlin J, Sánchez-Sampedro L, Del Pozo HP, Hernández CP, Marletta AS, De Lucrezia D, Sánchez-Fernández G, Fraile S, de Lorenzo V. 2020. SEVA 3.0: an update of the standard European vector architecture for enabling portability of genetic constructs among diverse bacterial hosts. Nucleic Acids Res 48:D1164–D1170. doi:10.1093/nar/gkaa114 31740968 PMC7018797

[33] Parales RE, Harwood CS. 1993. Construction and use of a new broad-host-range lacZ transcriptional fusion vector, pHRP309, for gram- bacteria. Gene 133:23–30. doi:10.1016/0378-1119(93)90220-w 8224891

[34] Herrero M, de Lorenzo V, Timmis KN. 1990. Transposon vectors containing non-antibiotic resistance selection markers for cloning and stable chromosomal insertion of foreign genes in gram-negative bacteria. J Bacteriol 172:6557–6567. doi:10.1128/jb.172.11.6557-6567.1990 2172216 PMC526845

[35] Miller JH. 1992. A short course in bacterial genetics. Cold Spring Harbor Laboratory Press, Plainview, N.Y.

[36] Ghosh S, Chan C-KK. 2016. Analysis of RNA-Seq data using TopHat and cufflinks. Methods Mol Biol 1374:339–361. doi:10.1007/978-1-4939-3167-5_18 26519415

[37] Livak KJ, Schmittgen TD. 2001. Analysis of relative gene expression data using real-time quantitative PCR and the 2^−ΔΔC_T_^ method. Methods 25:402–408. doi:10.1006/meth.2001.1262 11846609

[38] Lowden MJ, Skorupski K, Pellegrini M, Chiorazzo MG, Taylor RK, Kull FJ. 2010. Structure of Vibrio cholerae ToxT reveals a mechanism for fatty acid regulation of virulence genes. Proc Natl Acad Sci U S A 107:2860–2865. doi:10.1073/pnas.0915021107 20133655 PMC2840316

[39] Egan SM. 2002. Growing repertoire of AraC/XylS activators. J Bacteriol 184:5529–5532. doi:10.1128/JB.184.20.5529-5532.2002 12270809 PMC139625

[40] Charlier D, Bervoets I. 2019. Regulation of arginine biosynthesis, catabolism and transport in Escherichia coli. Amino Acids 51:1103–1127. doi:10.1007/s00726-019-02757-8 31267155

[41] Satchell KJF. 2015. Multifunctional-autoprocessing repeats-in-toxin (MARTX) toxins of Vibrios. Microbiol Spectr 3. doi:10.1128/microbiolspec.VE-0002-2014 PMC4509488 26185092

[42] Weber B, Croxatto A, Chen C, Milton DL. 2008. RpoS induces expression of the Vibrio anguillarum quorum-sensing regulator VanT. Microbiology (Reading) 154:767–780. doi:10.1099/mic.0.2007/014167-0 18310023

[43] Wang S, Payne GF, Bentley WE. 2020. Quorum sensing communication: molecularly connecting cells, their neighbors, and even devices. Annu Rev Chem Biomol Eng 11:447–468. doi:10.1146/annurev-chembioeng-101519-124728 32168999

[44] Schleif R. 2010. AraC protein, regulation of the l-arabinose operon in Escherichia coli, and the light switch mechanism of AraC action. FEMS Microbiol Rev 34:779–796. doi:10.1111/j.1574-6976.2010.00226.x 20491933

[45] Hulbert RR, Taylor RK. 2002. Mechanism of ToxT-dependent transcriptional activation at the Vibrio cholerae tcpA promoter. J Bacteriol 184:5533–5544. doi:10.1128/JB.184.20.5533-5544.2002 12270810 PMC139592

[46] Withey JH, DiRita VJ. 2006. The toxbox: specific DNA sequence requirements for activation of Vibrio cholerae virulence genes by ToxT. Mol Microbiol 59:1779–1789. doi:10.1111/j.1365-2958.2006.05053.x 16553883

[47] Zhang A, Rosner JL, Martin RG. 2008. Transcriptional activation by MarA, SoxS and rob of two tolC promoters using one binding site: a complex promoter configuration for tolC in Escherichia coli. Mol Microbiol 69:1450–1455. doi:10.1111/j.1365-2958.2008.06371.x 18673442 PMC2574956

[48] Weber GG, Klose KE. 2011. The complexity of ToxT-dependent transcription in Vibrio cholerae. Indian J Med Res 133:201–206. 21415495 PMC3089052

[49] Alekshun MN, Levy SB. 1999. Alteration of the repressor activity of MarR, the negative regulator of the Escherichia coli marRAB locus, by multiple chemicals in vitro. J Bacteriol 181:4669–4672. doi:10.1128/JB.181.15.4669-4672.1999 10419969 PMC103602

[50] Semchyshyn H, Bagnyukova T, Lushchak V. 2005. Involvement of soxRS regulon in response of Escherichia coli to oxidative stress induced by hydrogen peroxide. Biochemistry (Mosc) 70:1238–1244. doi:10.1007/s10541-005-0253-6 16336183

[51] Lindell K, Fahlgren A, Hjerde E, Willassen N-P, Fällman M, Milton DL. 2012. Lipopolysaccharide o-antigen prevents phagocytosis of Vibrio anguillarum by rainbow trout (Oncorhynchus mykiss) skin epithelial cells. PLoS One 7:e37678. doi:10.1371/journal.pone.0037678 22662189 PMC3360773

[52] Fridman CM, Keppel K, Gerlic M, Bosis E, Salomon D. 2020. A comparative genomics methodology reveals a widespread family of membrane-disrupting T6SS effectors. Nat Commun 11:1085. doi:10.1038/s41467-020-14951-4 32109231 PMC7046647

[53] Salomon D, Kinch LN, Trudgian DC, Guo X, Klimko JA, Grishin NV, Mirzaei H, Orth K. 2014. Marker for type VI secretion system effectors. Proc Natl Acad Sci U S A 111:9271–9276. doi:10.1073/pnas.1406110111 24927539 PMC4078801

[54] Schulze A, Mitterer F, Pombo JP, Schild S. 2021. Biofilms by bacterial human pathogens: clinical relevance - development, composition and regulation - therapeutical strategies. Microb Cell 8:28–56. doi:10.15698/mic2021.02.741 33553418 PMC7841849

[55] Roux FL, Wegner KM, Baker-Austin C, Vezzulli L, Osorio CR, Amaro C, Ritchie JM, Defoirdt T, Destoumieux-Garzón D, Blokesch M, Mazel D, Jacq A, Cava F, Gram L, Wendling CC, Strauch E, Kirschner A, Huehn S. 2015. The emergence of 761 Vibrio pathogens in Europe: ecology, evolution, and pathogenesis (Paris, 11– 762 12th March 2015). Front. Microbiol 6:830. doi:10.3389/fmicb.2015.00830 26322036 PMC4534830

[56] Singh RP, Kumari K. 2023. Bacterial type VI secretion system (T6SS): an evolved molecular weapon with diverse functionality. Biotechnol Lett 45:309–331. doi:10.1007/s10529-023-03354-2 36683130

[57] Bellair M, Withey JH. 2008. Flexibility of Vibrio cholerae ToxT in transcription activation of genes having altered promoter spacing. J Bacteriol 190:7925–7931. doi:10.1128/JB.00512-08 18849430 PMC2593237

[58] Withey JH, Dirita VJ. 2005. Vibrio cholerae ToxT independently activates the divergently transcribed aldA and tagA genes. J Bacteriol 187:7890–7900. doi:10.1128/JB.187.23.7890-7900.2005 16291662 PMC1291258

[59] Hsiao A, Toscano K, Zhu J. 2008. Post-transcriptional cross-talk between pro- and anti-colonization pili biosynthesis systems in Vibrio cholerae. Mol Microbiol 67:849–860. doi:10.1111/j.1365-2958.2007.06091.x 18179420

[60] Hsiao Ansel, Xu X, Kan B, Kulkarni RV, Zhu J. 2009. Direct regulation by the Vibrio cholerae regulator ToxT to modulate colonization and anticolonization pilus expression. Infect Immun 77:1383–1388. doi:10.1128/IAI.01156-08 19168737 PMC2663140

[61] Shi J, Wang F, Li F, Wang L, Xiong Y, Wen A, Jin Y, Jin S, Gao F, Feng Z, Li J, Zhang Y, Shang Z, Wang S, Feng Y, Lin W. 2022. Structural basis of transcription activation by Rob, a pleiotropic AraC/XylS family regulator. Nucleic Acids Res 50:5974–5987. doi:10.1093/nar/gkac433 35641097 PMC9178005

[62] Di Martino ML, Falconi M, Micheli G, Colonna B, Prosseda G. 2016. The multifaceted activity of the VirF regulatory protein in the Shigella lifestyle. Front Mol Biosci 3:61. doi:10.3389/fmolb.2016.00061 27747215 PMC5041530

[63] Stone JB, Withey JH. 2021. H-NS and ToxT inversely control cholera toxin production by binding to overlapping DNA sequences. J Bacteriol 203:e0018721. doi:10.1128/JB.00187-21 34228499 PMC8378478

[64] Kahramanoglou C, Webster CL, El-Robh MS, Belyaeva TA, Busby SJW. 2006. Mutational analysis of the Escherichia coli melR gene suggests a two-state concerted model to explain transcriptional activation and repression in the melibiose operon. J Bacteriol 188:3199–3207. doi:10.1128/JB.188.9.3199-3207.2006 16621812 PMC1447455

[65] Croxatto A, Chalker VJ, Lauritz J, Jass J, Hardman A, Williams P, Cámara M, Milton DL. 2002. VanT, a homologue of Vibrio harveyi LuxR, regulates serine, metalloprotease, pigment, and biofilm production in Vibrio anguillarum. J Bacteriol 184:1617–1629. doi:10.1128/JB.184.6.1617-1629.2002 11872713 PMC134878

[66] Milton DL, Hardman A, Camara M, Chhabra SR, Bycroft BW, Stewart GS, Williams P. 1997. Quorum sensing in Vibrio anguillarum: characterization of the vanI/vanR locus and identification of the autoinducer N-(3-oxodecanoyl)-L-homoserine lactone. J Bacteriol 179:3004–3012. doi:10.1128/jb.179.9.3004-3012.1997 9139920 PMC179066

[67] Milton DL. 2006. Quorum sensing in Vibrios: complexity for diversification. Int J Med Microbiol 296:61–71. doi:10.1016/j.ijmm.2006.01.044 16487746

[68] Ma L, Chen J, Liu R, Zhang X-H, Jiang Y-A. 2009. Mutation of rpoS gene decreased resistance to environmental stresses, synthesis of extracellular products and virulence of Vibrio anguillarum. FEMS Microbiol Ecol 70:130–136. doi:10.1111/j.1574-6941.2009.00713.x 19527291

[69] Defoirdt T. 2018. Quorum-sensing systems as targets for antivirulence therapy. Trends Microbiol 26:313–328. doi:10.1016/j.tim.2017.10.005 29132819

[70] Mauritzen JJ, Søndberg E, Kalatzis PG, Roager L, Gram L, Svenningsen SL, Middelboe M. 2023. Strain‐specific quorum‐sensing responses determine virulence properties in Vibrio anguillarum. Environ Microbiol 25:1344–1362. doi:10.1111/1462-2920.16356 36807464

[71] Bueno E, Pinedo V, Cava F. 2020. Adaptation of Vibrio cholerae to hypoxic environments. Front. Microbiol 11:528438. doi:10.3389/fmicb.2020.00739 PMC7212424 32425907

[72] Wasilko NP, Ceron JS, Baker ER, Cecere AG, Wollenberg MS, Miyashiro TI. 2021. Vibrio fischeri imports and assimilates sulfate during symbiosis with Euprymna scolopes. Mol Microbiol 116:926–942. doi:10.1111/mmi.14780 34212439 PMC8514163

[73] Friedrich CG, Bardischewsky F, Rother D, Quentmeier A, Fischer J. 2005. Prokaryotic sulfur oxidation. Curr Opin Microbiol 8:253–259. doi:10.1016/j.mib.2005.04.005 15939347

[74] Pinto R, Tang QX, Britton WJ, Leyh TS, Triccas JA. 2004. The Mycobacterium tuberculosis cysD and cysNC genes form a stress-induced operon that encodes a tri-functional sulfate-activating complex. Microbiology (Reading) 150:1681–1686. doi:10.1099/mic.0.26894-0 15184554

[75] Souto A, Montaos MA, Rivas AJ, Balado M, Osorio CR, Rodríguez J, Lemos ML, Jiménez C. 2012. Structure and biosynthetic assembly of piscibactin, a siderophore from Photobacterium damselae subsp. piscicida, predicted from genome analysis . Eur J Org Chem 2012:5693–5700. doi:10.1002/ejoc.201200818

[76] Wu G, Morris SM. 1998. Arginine metabolism: nitric oxide and beyond. Biochem J 336 (Pt 1):1–17. doi:10.1042/bj3360001 9806879 PMC1219836

[77] Mikkelsen H, Hui K, Barraud N, Filloux A. 2013. The pathogenicity island encoded PvrSR/RcsCB regulatory network controls biofilm formation and dispersal in Pseudomonas aeruginosa PA14. Mol Microbiol 89:450–463. doi:10.1111/mmi.12287 23750818 PMC3842833

[78] Banda MM, Pérez-Morales D, Zavala-Alvarado C, Nava-Galeana J, Bustamante VH. 2022. Two additional connections between the transcriptional programs controlling invasion and intracellular replication of Salmonella: HilD-SprB positively regulates phoP and slyA J Bacteriol 204:e0020422. doi:10.1128/jb.00204-22 36214553 PMC9664945

