# Supplementary material for: Remodulation of bacterial transcriptome after acquisition of foreign DNA: the case of irp-HPI high-pathogenicity island in Vibrio anguillarum
Source: mSphere. 2023 Dec 11;9(1):e00596-23. doi: 10.1128/msphere.00596-23 (PMC10826351; doi:10.1128/msphere.00596-23)
Supplement: Supplemental tables and figure — Tables S1 to S3 and Figure S1. [file msphere.00596-23-s0001.docx]

Remodulation of bacterial transcriptome after acquisition of foreign DNA: the case of *irp*-HPI high-pathogenicity island in *Vibrio anguillarum*

Marta A. Lages^1^, Ana do Vale^2^, Manuel L. Lemos^1^, and Miguel Balado^1*^

^1^ Department of Microbiology and Parasitology, Institute of Aquaculture, University of Santiago de Compostela, Santiago de Compostela, Spain.

^2^ Fish Immunology and Vaccinology Group, i3S-Instituto de Investigação e Inovação em Saúde, Universidade do Porto, Porto, Portugal.

**SUPPLEMENTARY MATERIAL**

Table S1. Primers used in this study.

Table S2. Strains and plasmids used in this study.

Table S3. Reads mapped statistics.

Table S4. Expression dataset. Excel Data Sheet under separate file.

Figure S1. SDS-PAGE gel of the fractions obtained during PbtA, PbtA^N^ and PbtA^C^ purification process.

Table S1. Primers used in this study. Restriction sites are underlined.

| Oligonucleotide  (5' -> 3') | |  | Amplicon size (bp) |
| --- | --- | --- | --- |
|  | **Protein expression** |  |  |
| PbtA | |  |  |
|  | AraC1_NdeI_F | GCCATATGGTATCTAAAATGAATCG | 990 |
|  | AraC1_XhoI_R | CCGCTCGAGGGGACGTTGACGATGTTTCC |  |
| PbtA^N^ | |  |  |
|  | AraC1_NdeI_F | GCCATATGGTATCTAAAATGAATCG | 645 |
|  | NTD_R_XhoI_R | GCGCTCGAGCTCACTGTCTTTTTTGTTGC |  |
| PbtA^C^ | |  |  |
|  | CTD_F_NdeI_Histag_NTD | CGCCATATGCACCACCACCACCACCACTCATCCCTTTCCAGTCGAAC | 342 |
|  | AraC1_XhoI_R_stop | CCGCTCGAGTTAGGGACGTTGACGATGTTTCC |  |
|  | ***irp*-HPI EMSA** |  |  |
| P*pbtA* | |  |  |
|  | AraC1_EMSA_F | TTCTTCCCCTAAAAAATGAC | 330 |
|  | AraC1_EMSA_R | TTTAGATACCATTCAAAAAT |  |
| P*frpA* (Probe 1) | |  |  |
|  | FrpA_1_EMSA_F | CAGGGTGCTCTCACGCCTAA | 333 |
|  | FrpA_EMSA_R | CGAATCTGTTTTCCTGTGGT |  |
| P*frpC* (Probe 9) | |  |  |
|  | FrpBC_EMSA_F | AATAAAGCTCCATAAATGGA | 340 |
|  | FrpBC_8_EMSA_R | TTTTGCGGCTCTTTTTTATA |  |
| Probe 2 | |  |  |
|  | FrpA_1_EMSA_F | CAGGGTGCTCTCACGCCTAA | 184 |
|  | FrpA_3_EMSA_R | GAACTTAATTAAAATTAAGC |  |
| Probe 3 | |  |  |
|  | FrpA_4_EMSA_F | AAAAATAGACGACCCGATCT | 149 |
|  | FrpA_EMSA_R | CGAATCTGTTTTCCTGTGGT |  |
| Probe 4 | |  |  |
|  | FrpA_3_EMSA_F | TAAAACAAAACCAGAAAATA | 64 |
|  | FrpA_3_EMSA_R | GAACTTAATTAAAATTAAGC |  |
| Probe 5 | |  |  |
|  | FrpA_4_EMSA_F | AAAAATAGACGACCCGATCT | 136 |
|  | FrpA_5_EMSA_R | CTGTGGTATCCATATTGAAC |  |
| Probe 6 | |  |  |
|  | FrpA_7_EMSA_F | ATTCAAGCTTGTTTAAAATA | 116 |
|  | FrpA_5_EMSA_R | CTGTGGTATCCATATTGAAC |  |
| Probe 7 | |  |  |
|  | FrpA_4_EMSA_F | AAAAATAGACGACCCGATCT | 116 |
|  | FrpA_7_EMSA_R | TTATAGTGCCGATTCTGTCT |  |
| Probe 8 | |  |  |
|  | FrpBC_EMSA_F | AATAAAGCTCCATAAATGGA | 760 |
|  | FrpBC_EMSA_1_R | ATACATCTTCGTAGACAGGG |  |
| Probe 10 | |  |  |
|  | FrpBC_EMSA_F | AATAAAGCTCCATAAATGGA | 220 |
|  | FrpBC_10_EMSA_R | AAGGCGCGCTTAAATTGGTT |  |
| Probe 11 | |  |  |
|  | FrpBC_EMSA_F | AATAAAGCTCCATAAATGGA | 160 |
|  | FrpBC_11_EMSA_R | TATTGAGCAATTGAGGATTA |  |
| Probe 12 | |  |  |
|  | FrpBC_14_EMSA_F | CATAAATGGAGGCTATTTTT | 150 |
|  | FrpBC_11_EMSA_R | TATTGAGCAATTGAGGATTA |  |
| Probe 13 | |  |  |
|  | FrpBC_13_EMSA_F | GGCTATTTTTGCTGAATATG | 140 |
|  | FrpBC_11_EMSA_R | TATTGAGCAATTGAGGATTA |  |
| Probe 14 | |  |  |
|  | FrpBC_EMSA_F | AATAAAGCTCCATAAATGGA | 140 |
|  | FrpBC_15_EMSA_R | ATGAATGTGGCCACTAATAT |  |
|  | **Genome EMSA** |  |  |
| P1 | |  |  |
|  | T6SS1_EMSA_P1_F | GCTGGTAAGATGAGTGAGGA | 523 |
|  | T6SS1_EMSA_P1_R | AGGAGCTACACTTCCTTCTT |  |
| P2 | |  |  |
|  | T6SS1_EMSA_P2_F | GCATATGTCGAAGAGTTGCT | 506 |
|  | T6SS1_EMSA_P2_R | CAGCGCCGTTAAGCCAGATT |  |
| P3 | |  |  |
|  | VanT_EMSA_F | GCGAGCTAAGTCTGCCATGA | 623 |
|  | VanT_EMSA_R | TGCAAACACTTCAAGGGCGA |  |
| P4 | |  |  |
|  | LPS_EMSA_F | GGATGTTTCTAACCATGGGG | 500 |
|  | LPS_EMSA_R | ACCTGATGTAGCAACAGCGC |  |
| P5 | |  |  |
|  | Hcp_EMSA_P1_F | GAGAGTGAGGGTTTAGACCA | 464 |
|  | Hcp_EMSA_P1_R | CGCATCACCGATTGAATCAG |  |
| P6 | |  |  |
|  | Hcp_EMSA_P2_F | ACCTTCAACGAAAGCATCGC | 519 |
|  | Hcp_EMSA_P2_R | GTGGATCGCACTGGAGACTT |  |
|  | **RT-qPCR** |  |  |
| 16S | |  |  |
|  | 16S_F_RTqPCR | ACCTCGGAACCGCATTTGAA | 225 |
|  | 16S_R_RTqPCR | CTCAAGGCCACAACCTCCAA |  |
| *frpA* | |  |  |
|  | FrpA_F_RTqPCR | CCTACCAAGTCAGCAGAAAG | 207 |
|  | FrpA_R_RTqPCR | ACGATACGTTTGAGCTGAGG |  |
| *vanT* | |  |  |
|  | VanT_F_RTqPCR | GTATGGTTTGAATGGAGCGC | 216 |
|  | VanT_R_RTqPCR | GGCTTCGTCTTGTATGCGAT |  |
| *hcp1* | |  |  |
|  | Hcp1_F_RTqPCR | GACCACGTTGTAACTGTACC | 240 |
|  | Hcp1_R_RTqPCR | GTGAATGTCCACGATAGAGG |  |
| *wza* | |  |  |
|  | Wza_F_RTqPCR | CGCAGATAAAGGCTGAGAGA | 252 |
|  | Wza_R_RTqPCR | GAAGTGTCTCTAGCTCCTGT |  |
| **PbtA^N^ mutant construction** | | |  |
|  | PbtA_1_F_XbaI | CCGTCTAGAGGATGTCCCACAACTGTTTC | 631 |
|  | PbtA_2_R_BamHI | GCGGGATCCCGTATGGATGTGGAGTTTGG |  |
|  | PbtA_3_F_BamHI | GGCGGATCCCGGATACGCAAAGCACACTC | 595 |
|  | PbtA_4_R_XhoI | CGGCTCGAGGCCATTAGTTTGCATGGACT |  |

Table S2. Strains and plasmids used in this study.

| **Strain** | **Relevant characteristics** | **Source** |
| --- | --- | --- |
| ***V. anguillarum*** | | |
| RV22 | Wild-type serotype O2 strain isolated from diseased turbot (Spain) | (26) |
| ML175 | RV22 with in-frame deletion of *pbtA* gene | This study |
| ML249 | ML175 carrying pML247 | This study |
| MB286 | RV22 carrying pMB276 | (16) |
| MB288 | RV22 carrying pMB277 | (16) |
| ML215 | RV22 carrying pML212 | This study |
| ML179 | RV22 with in-frame deletion *pbtA* carrying pMB276 | This study |
| ML181 | RV22 with in-frame deletion *pbtA* carrying pMB277 | This study |
| ML230 | RV22 with in-frame deletion *pbtA* carrying pML212 | This study |
| ML1308 | RV22 with in-frame deletion *pbtA^N^* carrying pMB276 | This study |
| ML1310 | RV22 with in-frame deletion *pbtA^N^* carrying pMB277 | This study |
| ML1312 | RV22 with in-frame deletion *pbtA^N^* carrying pML212 | This study |
| ***E. coli*** |  |  |
| BL21 | F– ompT gal dcm lon hsdSB(rB– mB–) [malB+]K-12(λS) | Laboratory strain |
| BL21 pLysS | F– ompT gal dcm lon hsdSB(rB–mB–) λ(DE3 [lacI lacUV5-T7p07 ind1 sam7 nin5]) [malB+]K-12(λS) | Laboratory strain |
| DH5α | Cloning strain | Laboratory strain |
| S17-1- *λpir* | RP4 (Km::Tn7, Tc::Mu-1) *pro-82 λpir recA1 end A1 thiE1 hsdR17 creC510* | (34) |
| **Plasmids** |  |  |
| pET20b(+) | Expression vector |  |
| pWKS30 | Low-copy cloning vector | (30) |
| pNidKan | Suicide vector derived from pCVD442 | (31) |
| pSEVA651 | mob Gm^r^ | (32) |
| pHRP309 | Low-copy number *lacZ* reporter plasmid, *mob* Gm^r^ | (33) |
| pML118 | S17 λpir pCar109 1-4 *pbtA* | (24) |
| pML247 | *pbtA* cloned in pSEVA651 | (24) |
| pML1287 | S17-1- *λpir* pCar109 1-4 *pbtA^N^* | This study |
| pMB276 | *frpA* promoter (*PfrpA*) fused to promoterless *lacZ* gene in pHRP309 | (16) |
| pMB277 | *pbtA* promoter (*PpbtA*) fused to promoterless *lacZ* gene in pHRP309 | (16) |
| pML212 | *frpC* promoter (*PfrpC*) fused to promoterless *lacZ* gene in pHRP309 | (25) |

Table S3. Reads mapped statistics.

| Sample | SRA study id. | Input reads | Reads mapped (%) | Multiple alignments reads (%) |
| --- | --- | --- | --- | --- |
| Wild type (sample A) | SRR25380061 | 23882118 | 19137111 (80.1%) | 174156 (0.9%) |
| Wild type (sample B) | SRR25380060 | 28469912 | 19520666 (68.6%) | 194412 (1.0%) |
| Wild type (sample C) | SRR25380059 | 27063524 | 23632512 (87,3%) | 511877 (2.2%) |
| *pbtA* mutant (sample A) | SRR25380058 | 24104387 | 20786307 (86.2%) | 121964 (0.6%) |
| *pbtA* mutant (sample B) | SRR25380057 | 24514810 | 20375881 (83.1%) | 30825 (0.2%) |
| *pbtA* mutant (sample C) | SRR25380056 | 27848110 | 25541908 (91.7%) | 48622 (0.2%) |


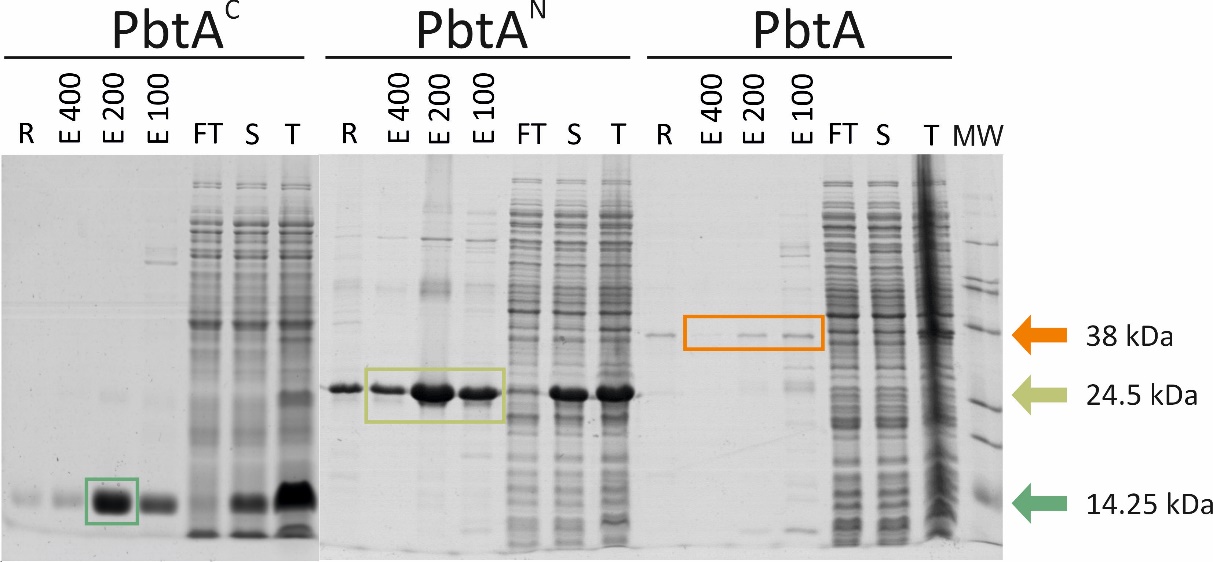


Figure S1. SDS-PAGE gel of the total fraction (T), soluble fraction (S), flow through (FT), elution with 100 mM imidazole (E100), elution with 200 mM imidazole (E200), elution with 400 mM imidazole (E400) and resin (R) of PbtA, PbtA^N^ and PbtA^C^ obtained during the purification process. The elutions highlighted in a rectangle were used for further protein concentration. Molecular-weight size marker (MW) consist of bands at 120, 100, 80, 60, 40, 30, 20 and 12 kD.
